# Quantification of biochemical PSA dynamics after radioligand therapy with [^177^Lu]Lu-PSMA-I&T using a population pharmacokinetic/pharmacodynamic model

**DOI:** 10.1186/s40658-024-00642-2

**Published:** 2024-04-24

**Authors:** Hinke Siebinga, Berlinda J. de Wit-van der Veen, Daphne M. V. de Vries-Huizing, Wouter V. Vogel, Jeroen J. M. A. Hendrikx, Alwin D. R. Huitema

**Affiliations:** 1 Department of Pharmacy and Pharmacology, The Netherlands Cancer Institute: Antoni Van Leeuwenhoek, Plesmanlaan 121, 1066 CX Amsterdam, The Netherlands; 2 Department of Nuclear Medicine, The Netherlands Cancer Institute: Antoni Van Leeuwenhoek, Amsterdam, The Netherlands; 3 Department of Radiation Oncology, The Netherlands Cancer Institute: Antoni Van Leeuwenhoek, Amsterdam, The Netherlands; 4 Department of Clinical Pharmacy, University Medical Center Utrecht, Utrecht University, Utrecht, The Netherlands; 5 Department of Pharmacology, Princess Máxima Center for Pediatric Oncology, Utrecht, The Netherlands

**Keywords:** ^177^Lu-PSMA-I&T, Population pharmacokinetic model, PKPD, PSA response, NONMEM, Prostate cancer

## Abstract

**Background:**

There is an unmet need for prediction of treatment outcome or patient selection for [^177^Lu]Lu-PSMA therapy in patients with metastatic castration-resistant prostate cancer (mCRPC). Quantification of the tumor exposure–response relationship is pivotal for further treatment optimization. Therefore, a population pharmacokinetic (PK) model was developed for [^177^Lu]Lu-PSMA-I&T using SPECT/CT data and, subsequently, related to prostate-specific antigen (PSA) dynamics after therapy in patients with mCRPC using a pharmacokinetic/pharmacodynamic (PKPD) modelling approach.

**Methods:**

A population PK model was developed using quantitative SPECT/CT data (406 scans) of 76 patients who received multiple cycles [^177^Lu]Lu-PSMA-I&T (± 7.4 GBq with either two- or six-week interval). The PK model consisted of five compartments; central, salivary glands, kidneys, tumors and combined remaining tissues. Covariates (tumor volume, renal function and cycle number) were tested to explain inter-individual variability on uptake into organs and tumors. The final PK model was expanded with a PD compartment (sequential fitting approach) representing PSA dynamics during and after treatment. To explore the presence of a exposure–response relationship, individually estimated [^177^Lu]Lu-PSMA-I&T tumor concentrations were related to PSA changes over time.

**Results:**

The population PK model adequately described observed data in all compartments (based on visual inspection of goodness-of-fit plots) with adequate precision of parameters estimates (< 36.1% relative standard error (RSE)). A significant declining uptake in tumors (k_14_) during later cycles was identified (uptake decreased to 73%, 50% and 44% in cycle 2, 3 and 4–7, respectively, compared to cycle 1). Tumor growth (defined by PSA increase) was described with an exponential growth rate (0.000408 h^−1^ (14.2% RSE)). Therapy-induced PSA decrease was related to estimated tumor concentrations (MBq/L) using both a direct and delayed drug effect. The final model adequately captured individual PSA concentrations after treatment (based on goodness-of-fit plots). Simulation based on the final PKPD model showed no evident differences in response for the two different dosing regimens currently used.

**Conclusions:**

Our population PK model accurately described observed [^177^Lu]Lu-PSMA-I&T uptake in salivary glands, kidneys and tumors and revealed a clear declining tumor uptake over treatment cycles. The PKPD model adequately captured individual PSA observations and identified population response rates for the two dosing regimens. Hence, a PKPD modelling approach can guide prediction of treatment response and thus identify patients in whom radioligand therapy is likely to fail.

**Supplementary Information:**

The online version contains supplementary material available at 10.1186/s40658-024-00642-2.

## Introduction

Most prostate cancer (PCa) cells show an overexpression of the prostate-specific membrane antigen (PSMA) receptor [1, 2]. Radioligands targeting this PSMA receptor proved effective agents for diagnosis and treatment in metastatic castration-resistant prostate cancer (mCRPC) [3]. Response to treatment is generally evaluated by assessing volume reduction on conventional-imaging and/or decrease in serum prostate-specific antigen (PSA) levels, while standardized response criteria for PSMA-based PET are not established yet [4, 5]. Though this debate on how to evaluate treatment response is ongoing, approximately 20–30% of all patients treated with Lutetium-177 (^177^Lu) prostate-specific membrane antigen ligand ([^177^Lu]Lu-PSMA) show no response to this expensive treatment independent of the response measure used [3, 6–9]. It remains unclear what causes this non-response, though many potential demographic, histological, biochemical and imaging factors have already been investigated to predict response to treatment [10, 11]. Results of these studies were inconsistent mainly due to the small and heterogeneous patient populations, which is further complicated by the fact that PSMA-therapy is adopted as “last line” treatment in a heavily pretreated population. Consequently, there still is an unmet need for early prediction of individual treatment outcome or better patient selection before initializing [^177^Lu]Lu-PSMA therapy.

The mechanism of action of [^177^Lu]Lu-PSMA is the result of a cytotoxic radiation dose due to β minus particle (β^−^) emission, which modifies the tumor microenvironment and induces DNA damage followed by cell death [12]. Theoretically, a higher (homogeneous) cumulative absorbed dose (i.e. the energy deposited by the ionizing radiation) in tumor lesions will provide a better response to treatment. Clinical and preclinical data demonstrated that the absorbed radiation dose in tumors is indeed correlated with cellular damage and treatment response [13–16]. Therefore, individual predictions of tumor absorbed doses could help to identify patients with a higher probability of responding to [^177^Lu]Lu-PSMA therapy. Still, this exposure–response relationship was not evidently assessed in a larger population yet, and it remains unknown whether this potential association is linear or a maximum effect is reached at some point [17, 18].

A pharmacokinetic/pharmacodynamic (PKPD) modelling approach was selected to assess the exposure–response relation of [^177^Lu]Lu-PSMA therapy in patients with mCRPC. The use of such mathematical models is well accepted for nonradioactive drugs to establish and describe dose-concentration–response relationships [19, 20]. In this work, PSA was used to describe treatment response, because any decline in serum PSA after [^177^Lu]Lu-PSMA therapy was predictive for response and in several studies a PSA-decline ≥ 50% was associated with a better progression free and overall survival [10, 21, 22]. Hence, a population PK model for [^177^Lu]Lu-PSMA-I&T was developed using post-administration SPECT/CT data and was extended to a PKPD model to describe individual serum PSA dynamics after therapy in patients with mCRPC. Using this model, we aimed to better understand the exposure–response relationship for [^177^Lu]Lu-PSMA-I&T and, ultimately, assess whether individual PSA dynamics could be related to individual ^177^Lu-accumulation profiles to aid patient selection.

## Materials and methods

### Patients and data collection

Data from patients treated in our hospital with [^177^Lu]Lu-PSMA-I&T between September 2019 and January 2023 were collected for population PK model development. This retrospective data collection was approved by the Institutional Review Board of the Netherlands Cancer Institute (IRBd21288). Baseline patient characteristics were collected, such as age, body weight, height and serum creatinine clearance. In addition, individual PSA concentrations were collected and used as input for the PD model. As pre-treatment PSA value, only the last observation prior to [^177^Lu]Lu-PSMA-I&T treatment (< 6 weeks) was included in the analysis, to avoid taking into account PSA changes due to other previous treatments. For the same reason, PSA observations after the last [^177^Lu]Lu-PSMA-I&T administration were collected up to start of a new treatment.

Administration of ~ 7.4 GBq [^177^Lu]Lu-PSMA-I&T was followed by 3-bedposition (top of head to mid-thighs) post-administration SPECT/CT imaging at 4, 24 h and 5–7 days post injection (or only at 24 h and 5–7 days post injection due to changes in the scan protocol in our hospital). Two different dosing schedules were clinically used in our hospital during the inclusion period; four cycles with a six-week interval and two cycles with a two-week interval repeated after twelve weeks based on initial response to therapy (‘4 × 6’ vs ‘2 × 2 – repeated after twelve weeks’). Quantification of radioligand uptake on post-administration SPECT-images in relevant tissues (i.e. kidneys and salivary glands) was performed using PLANET® Onco (DOSIsoft, SA) by contouring the entire kidney cortex and placing spherical volumes-of-interest in the parotid-submandibular gland (20 mm diameter). Blood data were also derived from SPECT-images by placing region-of-interests (20 mm diameter) in three to four consecutive slices in the descending aorta. For tumors, only uptake in target lesions with a diameter > 20 mm on CT was segmented (using 20 mm diameter spheres) to avoid a negative bias in quantified activity concentration in small volumes, with a maximum of five lesions per patient (two per organ system) [23]. Tumor volumes were determined for segmented (target) tumors based on diagnostic pre-treatment PSMA-PET/CT imaging using IntelliSpace Portal (Philips Healthcare, The Netherlands) with a semi-automatic threshold segmentation method of 45% of the maximum value of the standardized uptake value normalized to lean body mass (SUL_max_).

For model development, accumulation in the kidney, salivary gland and tumor compartments were lumped, by means that the kidney compartment represented both kidneys and the tumor compartment represented all tumor tissue. Whole-body data was not used in model development, because no whole body images were available from our clinical setting. Observations and predictions for the central compartment were in concentrations (MBq/L), whereas for salivary glands, kidneys and tumors the radioactivity amounts (MBq) were used. All radioactivity data were corrected for decay to the time of injection. Subsequently, radioactivity blood concentrations (MBq/L) were corrected using a previously determined correction factor for [^177^Lu]Lu-PSMA-617 in blood (linear regression with an intercept of 6.27 MBq/L and a slope of 0.828) [24]. This correction based on PSMA-617 data might not be optimal for PSMA-I&T, but no other correction factors were available for using blood data derived from SPECT scans in population PK models.

### Included patients and data

A total of 79 patients received at least one cycle of ~ 7.4 GBq [^177^Lu]Lu-PSMA-I&T in our hospital. All patients had PSMA-positive tumor lesions on pre-treatment diagnostic [^68^Ga]Ga-PSMA PET/CT, and no PSMA-negative tumor lesions as verified with diagnostic contrast-enhanced CT, according to the criteria of the VISION study [7]. Patients 1–10 received (up to) four cycles of ~ 7.4 GBq with an interval of six weeks, while all other patients received two cycles of ~ 7.4 GBq with an interval of two weeks. In this latter patient group, patients were eligible for extra treatment cycles in case of a PSA response based on the initial treatment series. Post-administration SPECT/CTs were acquired at 4.6 ± 0.95 h, 23.8 ± 4.5 h and 6.8 ± 0.46 days post injection. SPECT/CTs were unreliable in some patients due to inaccurate image reconstruction (n = 22 scans) and inaccurate registered scan times (n = 3 scans), thus these data were excluded from analysis. In addition, negative blood concentrations (after correction) at the first time points after injection were excluded from analysis (n = 17 data observation). Patients that exclusively had tumor lesions < 20 mm diameter (determined on diagnostic imaging) were excluded from analysis (n = 3 patients). This resulted in a total of 76 included patients, with 409 SPECT/CT scans available for PK model development. The median administered activity was 7.38 GBq (5.61–7.76 GBq) and patients received up to eight cycles of [^177^Lu]Lu-PSMA-I&T. A total of 566 PSA observations were available for development of the PD model, after exclusion of PSA concentrations collected once a new treatment succeeded [^177^Lu]Lu-PSMA-I&T (n = 6 patients). Patient characteristics are provided in Table 1.

**Table 1:** Patient characteristics

Characteristic	Median (range)
Patients (n)	76
Age (years)	73 (48–91)
Weight (kg)	79 (61–116)
GFR (calculated using Cockcroft Gault) (mL/min)	80.9 (25.9–181)
Hematocrit	0.35 (0.22–0.44)
Baseline PSA (µg/L)	260 (0.12–4896)
Tumor volume of segmented tumors (L)	0.0443 (0.000122–0.546)
Injected radioactivity (MBq)	7378 (5605–7763)
Received dosing schedule (n)	
‘4 × 6’	10
‘2 × 2—repeated after twelve weeks’	66
Number of cycles received (n)	
1	76
2	74
3	48
4	37
5	6
6	4
7	1
8	1

*GFR* glomerular filtration rate, *PSA* prostate-specific antigen

### Pharmacokinetic model development

Model development was informed by a previously developed population PK model for [^177^Lu]Lu-PSMA-617 [24]. The structural model consisted of five compartments, representing a central compartment, salivary glands, kidneys, tumor lesions and a combined remaining tissue compartment. Each compartment is a representation of the whole organ or tumor and no distinction between subcompartments was considered, because the exact location of radioactivity can also not be determined based on nuclear images. A top-down approach was used for this population PK model and most parameters were fitted rather than fixed based on prior knowledge or assumptions. Also, no further model selection (e.g. with single or multi-compartments per organ of interest) was performed, as is regularly done for PBPK models including parameter assumptions [25]. Renal excretion of [^177^Lu]Lu-PSMA-I&T was described by an excretion rate constant (k_10_) from the central compartment. Renal excretion was not added to the kidney compartment, because kidney exposure mainly reflects intracellular uptake rather than radioactivity located in the vascular part. Radiopharmaceutical transport between the central compartment and all other compartments was described by rate constant (k) parameters. Saturable binding equilibriums (maximum binding capacity (B_MAX_)) were tested for all PSMA-expressing compartments according to Eq. 1, whereas inter-individual (IIV) and inter-occasion variability (IOV) were added following Eq. 2. Residual unexplained variability (RUV) for the central compartment was described by a combined proportional and additive residual error model (Eq. 3) and by a proportional residual error model for all other compartments (Eq. 4). A combined error model for the central compartment prevented the model being highly reliant on the lowest blood observations (with relatively high noise). Proportional error models are commonly used for biokinetic data in organs [26]. Detailed information regarding testing saturable binding equilibriums for all compartments and addition of IIV, IOV and RUV was specified previously [24].

1 $$\frac{{dA_{target} }}{dt} = k_{in} *A_{central} *\left( {1 - \frac{{A_{target} }}{{B_{MAX} }}} \right) - k_{out} *A_{target}$$

where *k*_*in*_ and *k*_*out*_ represent the rate constants, *A*_*central*_ and *A*_*target*_ represent the compound amounts in the central and target compartment, respectively, and *B*_*MAX*_ is the maximum binding capacity in the target compartment (i.e. PSMA receptor expression).

2 $$P_{i} = P_{pop} *e^{{\eta_{i} }}$$

3 $$C_{obs,ij} = C_{pred, ij} *\left( {1 + \varepsilon_{p, ij} } \right) + \varepsilon_{add, ij}$$

4 $$C_{obs,ij} = C_{pred, ij} *\left( {1 + \varepsilon_{p, ij} } \right)$$

where *P* represents the PK parameter estimate for individual *i*, *P*_*pop*_ represents the population PK parameter estimate and *η* represents the IIV or IOV effect for individual *i* with mean 0 and variance *ω*^2^. *C*_*obs*_ represents the observed concentration for individual *i* and observation *j*, *C*_*pred*_ represents the predicted concentration and *ε*_*p*_ and *ε*_*add*_ represent the proportional and additive error, respectively, both distributed with mean 0 and variance *σ*^2^.

Covariate testing was performed based on clinical plausibility, to evaluate whether covariates could partly explain IIV on PK parameters. Renal function was assumed a relevant covariate for clearance, since [^177^Lu]Lu-PSMA-I&T is renally excreted [27]. The clinical plausibility of tumor volume being important for PSMA-ligand distribution was shown in several previous publications [24, 28–31]. Only target tumor volume was available for covariate modelling, and, therefore, total tumor volume could not be used. Similar to the previously developed model [24], the tested covariates were tumor volume on the salivary gland (k_12_), kidney (k_13_) and tumor uptake rate (k_14_) and renal function (by means of the estimated glomerular filtration rate based on creatinine clearance) on the excretion rate (k_10_, i.e. renal clearance from the blood). Tumor volume was tested as a power (Eq. 5), linear (Eq. 6) and exponential (Eq. 7) covariate function, whereas renal function was evaluated as a linear covariate (Eq. 6) [32]. A linear covariate model for renal function is pharmacologically most suitable, since renal excretion via glomerular filtration is the only route of elimination and this is expected to linearly increase with an increased estimated glomerular filtration rate.

5 $$P_{cov} = P_{pop} *\left( {\frac{COV}{{COV_{median} }}} \right)^{{\theta_{cov} }}$$

6 $$P_{cov} = P_{pop} + \left( {\theta_{cov} *\left( {\frac{COV}{{COV_{median} }}} \right)} \right)$$

7 $$P_{cov} = P_{pop} *e^{{\theta_{cov} *\left( {\frac{COV}{{COV_{median} }}} \right)}}$$

where *P*_*cov*_ is the estimated individual parameter value, *P*_*pop*_ is the estimated population parameter value, *COV* is the individual’s covariate value, *COV*_*median*_ is the median value of the tested covariate and *θ*_*cov*_ represents the estimated effect of the covariate on *P*_*pop*_.

In addition, body weight was tested on all PK parameters by means of allometric scaling in relation to the median body weight, where exponents for volume of distribution (V) and rate constants (k) were set to 1 and -0.25, respectively [33]. Lastly, for radionuclide therapies, there is an ongoing debate about the hypothesis that the absorbed dose in tumors is likely to decrease in later cycles. This phenomenon was shown by Garkavij et al*.* for [^177^Lu]Lu-DOTATATE, but clear evidence regarding this effect in PSMA-therapy is lacking [34]. To provide evidence regarding the existence of this cycle effect for [^177^Lu]Lu-PSMA-I&T, all cycles were tested as dichotomous covariates on uptake in salivary glands (k_12_), kidney (k_13_) and tumors (k_14_) by means of relating the uptake in a cycle to a fraction of the uptake in the first cycle, according to Eq. 8 [35]. Due to limited patients receiving more than four cycles, these cycles were lumped as a single additional cycle effect.

8 $$P_{cov} = P_{pop} * \theta_{cov1}^{cycle 2} * \theta_{cov2}^{cycle 3} *\theta_{cov3}^{cycle 4 - 7}$$

where *P*_*cov*_ is the estimated individual uptake parameter value, *P*_*pop*_ is the estimated population uptake parameter value and *θ*_*cov*_ values represent the fraction of uptake for that cycle compared to *P*_*pop*_ (i.e. uptake in the first cycle).

### Pharmacodynamic model development

A sequential modelling approach was used for PKPD model development [36], where an additional compartment was considered to describe PSA concentrations over time. Baseline PSA concentrations were estimated as a typical population value with an estimated IIV on this population baseline value. PSA growth was included using an exponential first-order PSA growth rate (k_G_), as previously described by Van Hasselt et al*.* [37]. The direct effect of [^177^Lu]Lu-PSMA-I&T treatment on PSA was modelled with E_drug_ being elimination from the PSA compartment. For E_drug_, a linear function, an E_max_ function and a sigmoid E_max_ model were evaluated (see Eqs. 9–11, respectively) [17].

9 $$E_{drug} = k_{D} * C_{tumor}$$

10 $$E_{drug} = \frac{{E_{MAX} * C_{tumor} }}{{EC_{50} + C_{tumor} }}$$

11 $$E_{drug} = \frac{{E_{MAX} * C_{tumor}^{\gamma } }}{{EC_{50}^{\gamma } + C_{tumor}^{\gamma } }}$$

where *k*_*D*_ is a drug-induced effect, *C*_*tumor*_ is the estimated concentration in the tumor compartment (taking into account radioactive decay), *E*_*MAX*_ is the maximal drug induced effect of [^177^Lu]Lu-PSMA-I&T on PSA, *EC*_*50*_ is the [^177^Lu]Lu-PSMA-I&T concentration at which half of the maximum drug-induced effect on PSA occurs and *γ* is a hill coefficient (that was both estimated and fixed during model development).

Some patients showed an ongoing decrease in PSA even after treatment with [^177^Lu]Lu-PSMA-I&T was completed and radioactivity in tumors was eliminated. Therefore, a delay of the PD effect after administration of [^177^Lu]Lu-PSMA-I&T (E_delayed_) was evaluated using an effect compartment with a linear drug effect (Eq. 8) on the PSA compartment [38]. This delayed effect was assessed both solely as well as additional to the direct effect (E_drug_). In the final model, both direct and a delayed effect were included, so that the total PSA dynamics over time were described following Eq. 12. No decrease of PSA was assumed without an initial treatment cycle.

12 $$\frac{dPSA}{{dt}} = k_{G} * PSA - (E_{drug} + E_{delayed} )*PSA$$

where *k*_*G*_ is the first-order PSA growth rate, *PSA* represents the PSA concentration at a certain time point, *E*_*drug*_ and *E*_*delayed*_ are functions representing the direct effect and delayed effect of [^177^Lu]Lu-PSMA-I&T on PSA, respectively.

Furthermore, covariates were tested to improve the model fit of the PD model, where selection of potential covariates was guided by clinical and biological relevance. Tumor volume and age were assumed to be related to PCa disease severity and thus were tested as covariates to describe variability on baseline PSA (by using power and linear functions, according to Eqs. 5 and 6, respectively). In addition, resistance of the [^177^Lu]Lu-PSMA-I&T effect over time and a change of k_G_ during treatment were assessed. IIV was evaluated for all PD parameters by using an exponential function (according to Eq. 2). In case the random effect distribution was non-normal (and thus visually deviated from the assumed distribution shape), a box-cox transformation of that specific *η* was performed [39]. A proportional (Eq. 4) and combined proportional and additive residual error model (Eq. 3) were tested to describe residual errors [32].

### Model evaluation

Evaluation of model fits were guided by physiological plausibility of parameters and evaluation of goodness-of-fit (GOF) plots [40], prediction corrected visual predictive checks (pcVPC) [41], the change in objective function value (dOFV), successful minimization, decrease in IIV and the uncertainty in parameter precision. These aspects together supported model evaluation and thereby determined whether the model adequately fit the observed data [40]. Precision of parameter estimates was determined using the sampling importance resampling (SIR) approach [42]. For hierarchical nested models, a *p* value < 0.05 was considered a significant improvement of the model fit (corresponding to a decrease in OFV of ≥ 3.84 points following a Chi-square distribution with 1 degree of freedom).

### Simulations

Population simulations based on the final PKPD model were performed to evaluate treatment response for both dosing regimens. A patient population was simulated (n = 2000) based on patient characteristic distributions of all included covariates of our clinical population (n = 76). The simulated dosing regimens included: four cycles of 7.4 GBq with a six-week interval (referred to as ‘4 × 6’) and two cycles of 7.4 GBq with a two-week interval which was repeated after 12 weeks (referred to as ‘2 × 2 − repeated after twelve weeks’). Endpoints were response and stable disease at the end of treatment (i.e., 24 weeks after start of treatment), where response was defined as a ≥ 50% decrease in PSA and stable disease as no increase in PSA [43, 44].

### Exposure thresholds

As an exploratory analysis, the final population PKPD model was used to assess a potential tumor exposure threshold required to achieve treatment response. The individually (Bayesian) estimated area under the curve (AUC) based on the first two treatment cycles was related to the change in PSA (%) for each patient. Since PSA is a continuous response measure and there is no defined moment to determine response, two endpoints for response were considered: nadir PSA and the last measured PSA value after the first two treatment cycles. A linear regression was performed to assess the relationship and was subsequently used to define tumor AUC thresholds required to achieve treatment response (≥ 50% decrease in PSA) [43, 44]. Formal statistical testing was not possible, since individual (Bayesian) estimates of tumor exposure cannot be considered independent variables.

### Software

The modelling was performed using NONMEM (version 7.5; ICON development Solutions, Ellicott City, MD) using the first-order conditional estimation method with interaction (FOCE-I) and ADVAN13. Data processing, visualization of GOF plots and pcVPCs and simulations based on the PKPD final model as well as the linear regression were performed using R (version 4.2.1).

## Results

### Population pharmacokinetic model

**Fig. 1:**
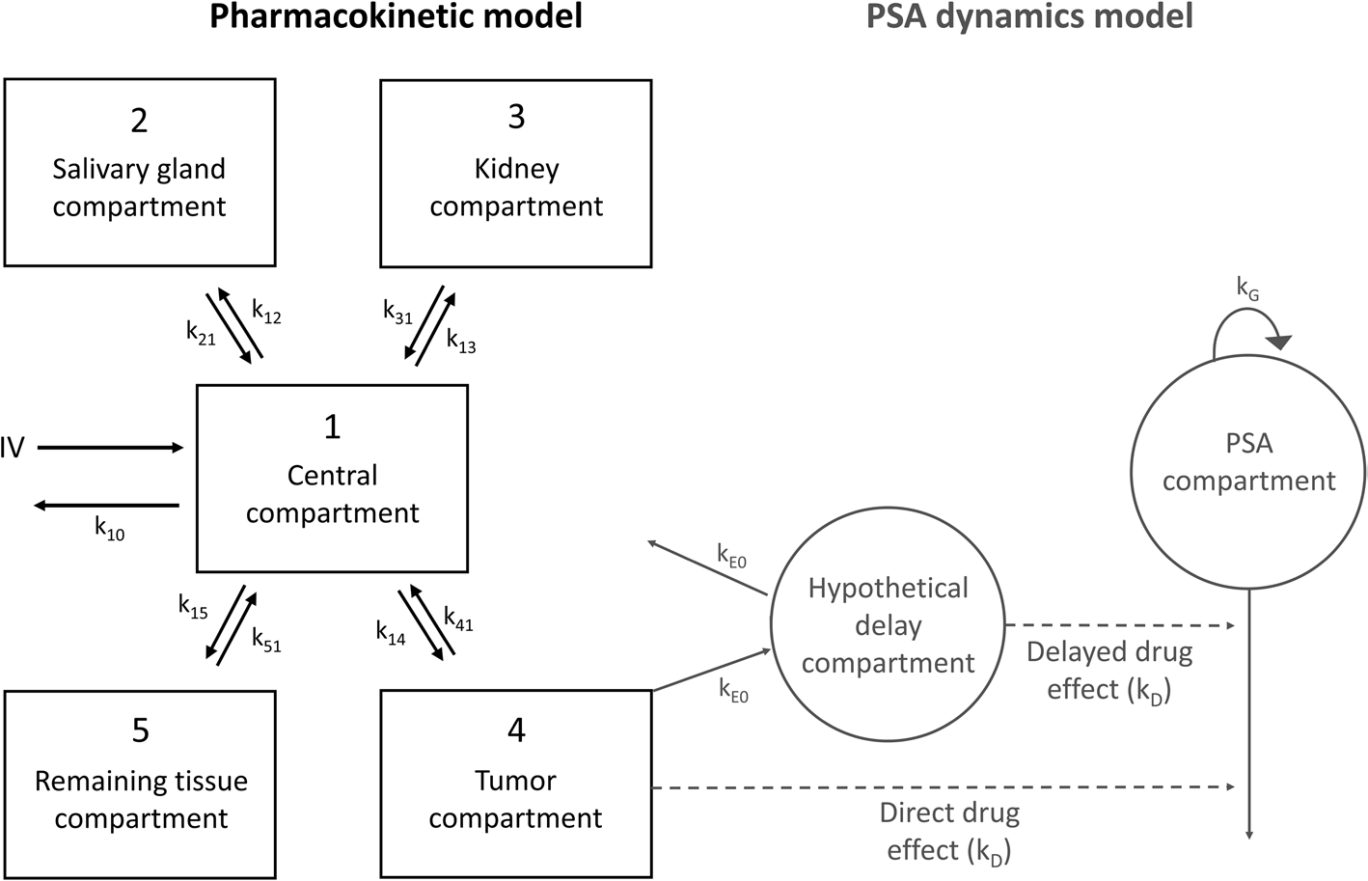
Schematic overview of the final PKPD model for [^177^Lu]Lu-PSMA-I&T describing PSA dynamics, where the PK and the PD model are depicted in black and grey, respectively

**Fig. 2:**
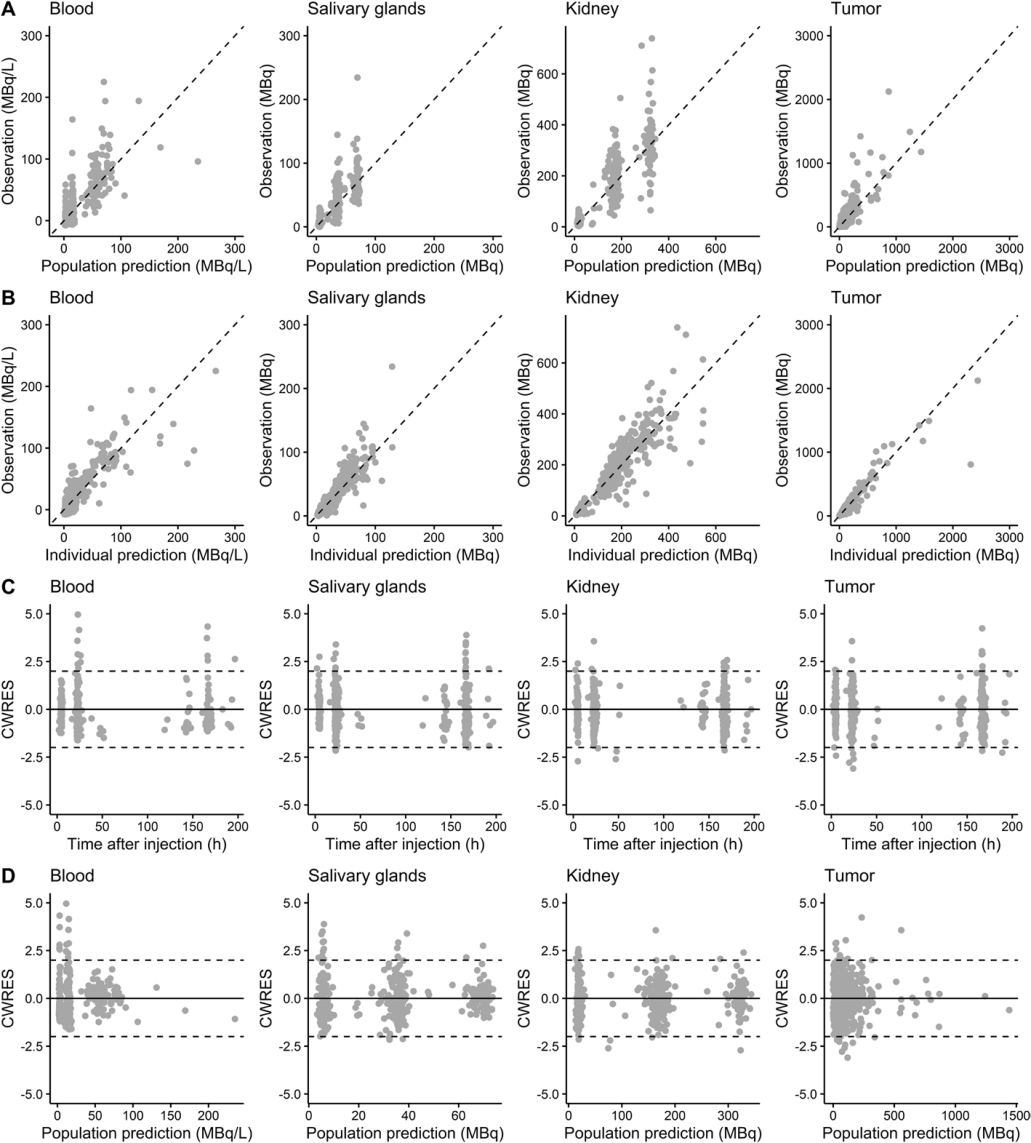
Goodness-of-fit plots of the final population PK model for [^177^Lu]Lu-PSMA-I&T, including population predictions (PRED) versus observations (**A**), individual predictions (IPRED) versus observations (**B**), conditional weighted residuals (CWRES) versus time after injection (**C**) and CWRES versus PRED (**D**), for all compartments separately

A five-compartment model with first-order kinetics was developed to describe the observed [^177^Lu]Lu-PSMA-I&T accumulation data. An overview of the population PK model (including the PD model) is provided in Fig. 1. Renal excretion (k_10_) was estimated 0.253 h^−1^ (5.2% RSE). The central volume of distribution (V1) was fixed to 10.3 L, because blood data derived from scans were considered too unreliable for accurate parameter estimation. Final model parameter estimates for k_in_ values to all compartments were 0.0105 h^−1^ (9.2% RSE), 0.0321 h^−1^ (6.9% RSE), 0.00967 h^−1^ (9.9% RSE) and 0.275 h^−1^ (6.8% RSE) for compartment two to five (i.e. salivary glands, kidneys, tumor and remaining tissue), respectively. Allometric scaling was added to all PK parameters (dOFV −11.3). As mentioned before, creatinine clearance, tumor volume and cycle effects were introduced as covariates. Serum creatinine clearance was not identified as a covariate on k_10_, since the OFV and model fit did not improve (dOFV −0.207). Tumor volume was selected as a covariate on the tumor uptake rate (k_14_) using a power function, which showed the best improvement in model fit (dOFV −89.9, compared to dOFV −87.3 for a linear function and dOFV −31.0 for an exponential function). The estimated covariate value was 1.08 (7.5% RSE), where a two-times higher volume resulted in a 2.11-fold increased tumor uptake rate. A slight tumor sink effect was identified for salivary glands, where an increased tumor volume resulted in a lower salivary gland uptake rate (k_12_) (using a power function with an estimated covariate value of 0.0910 (30.0% RSE), dOFV −7.46). No effect of tumor volume on the kidney uptake rate (k_13_) was identified (dOFV −1.53). A cycle effect was identified with a significant decreased [^177^Lu]Lu-PSMA-I&T uptake in tumors in later cycles (dOFV −157), where the tumor uptake rate decreased to 73%, 50% and 44% uptake in cycle 2, 3 and 4–7 compared to cycle 1, respectively. Unfortunately, the limited available data for the ‘4 × 6’ dosing scheme were not sufficient to identify different cycle-effects for both dosing schemes. Therefore, these differences in dosing intervals might have impacted our estimates and these estimates probably mainly reflect the ‘2 × 2 – repeated after twelve weeks’ scheme. Still, when excluding all patients receiving the ‘4 × 6’ scheme, a smaller decrease of 82.9% was estimated for cycle 2 (whereas other estimates did not substantially change). Saturable uptake into the salivary glands was included with an estimated B_MAX_ of 134 MBq (15.5% RSE). Adding saturable uptake for the kidney and tumor compartments resulted in non-identifiable B_MAX_ estimates, implying no detectable saturable binding with current specific activities, and thus were not included. Cycle-to-cycle variability (or IOV) was most profound for tumors (estimated 37.8% (16.5% RSE)) and was thus only added on the tumor uptake rate parameter for reasons of model simplicity. This IOV reflects a random variability in tumor uptake between cycles, in addition to the identified cycle effect (i.e. structural decrease in tumor uptake in subsequent cycles). IIV was highest on the tumor uptake rate parameter (k_14_) and B_MAX_ of the salivary gland compartment (63.4% and 67.0% coefficient of variation (CV), respectively), while IIV on other PK parameters was rather small (< 35% CV). All PK parameters were estimated with adequate precision (RSE < 36.1%). RUV for the central compartment was best explained by estimating the proportional (similar to our previous model [24]), but also the additive component (dOFV -40.4). Final population and individual predicted concentrations were in line with the observed [^177^Lu]Lu-PSMA-I&T data for all compartments (see Fig. 2). Final parameter estimates and parameter precision for the population PK model are provided in Table 2, whereas individual prediction vs observation plots are provided in Additional file 1: Figures S1-S3.

**Table 2:** Model estimates of the final pharmacokinetic model for [^177^Lu]Lu-PSMA-I&T

Pharmacokinetic parameters	Estimate (RSE%)	95% CI
*Structural parameters*		
k_10_ (h^−1^)	0.253 (5.2%)	0.229–0.281
k_12_ (h^−1^)	0.0105 (9.2%)	0.00878–0.0125
Tumor volume on k_12_^a^	0.0910 (30.0%)	0.0350–0.544
k_21_ (h^−1^)	0.0629 (6.3%)	0.0555–0.0715
k_13_ (h^−1^)	0.0321 (6.9%)	0.0281–0.0368
k_31_ (h^−1^)	0.0625 (5.4%)	0.0561–0.0689
k_14_ (h^−1^)	0.00967 (9.9%)	0.00807–0.0117
Tumor volume on k_14_^a^	1.08 (7.5%)	0.921–1.24
Cycle 2 on k_14_^b^	0.731 (8.1%)	0.619–0.857
Cycle 3 on k_14_^b^	0.498 (11.3%)	0.407–0.624
Cycle 4–7 on k_14_^b^	0.436 (11.6%)	0.350–0.544
k_41_ (h^−1^)	0.0150 (3.4%)	0.0141–0.0161
k_15_ (h^−1^)	0.275 (6.8%)	0.240–0.314
k_51_ (h^−1^)	0.0247 (6.5%)	0.0216–0.279
B_MAX_ compartment 2 (MBq)	134 (15.5%)	100–183
V1 (L)	10.3^c^	
*Inter-individual variability*		
k_10_ (CV%)	33.1 (18.5%)	27.6–39.7
k_13_ (CV%)	33.4 (22.2%)	26.8–40.8
k_14_ (CV%)	63.4 (18.9%)	52.0–75.1
k_15_ (CV%)	29.1 (35.5%)	20.2–40.0
B_MAX_ compartment 2 (CV%)	67.0 (36.1%)	49.1–94.2
*Inter-occasion variability*		
k_14_ (CV%)	37.8 (16.5%)	31.5–44.1
*Residual unexplained variability*		
Proportional error compartment 1 (CV%)	55.5 (29.0%)	39.0–71.6
Additive error compartment 1 (MBq/L)	9.57 (16.8%)	8.02–11.2
Proportional error compartment 2 (CV%)	39.7 (8.6%)	36.7–43.2
Proportional error compartment 3 (CV%)	31.9 (8.5%)	29.4–34.6
Proportional error compartment 4 (CV%)	32.7 (9.7%)	30.0–36.2

95% CI and RSE values were obtained from the SIR

*B*_*MAX*_ maximum binding capacity, *CI* confidence interval, *CV%* coefficient of variation, *RSE* relative standard error, *SIR* sampling importance resampling, *V1* central volume of distribution

^a^Added using a power covariate function: $P_{cov} = P_{pop} *\left( {\frac{COV}{{COV_{median} }}} \right)^{{\theta_{cov} }}$

^b^Added as fractional change: $P_{cov} = P_{pop} * \theta_{cov1}^{cycle 2} * \theta_{cov2}^{cycle 3} *\theta_{cov3}^{cycle 4 - 7}$

^c^Fixed parameter

### Population pharmacodynamic model

The PK model was expanded with one PD compartment representing PSA dynamics. The baseline PSA was fixed to avoid overparameterization and high correlations between parameter estimates. This value was initially estimated 140 µg/L based on baseline PSA measurements and fixed to this value during further model development, therefore it still reflects the baseline PSA of this population. Tumor volume was identified as a linear covariate on the baseline PSA with an estimated covariate value of 57.5 µg/L (38.9% RSE) (dOFV -4.51). Age did not explain IIV on the baseline PSA (dOFV -1.01). The exponential PSA growth rate was estimated 0.000408 h^−1^ (14.2% RSE) and this parameter was not identified to change during treatment. A linear model described the direct drug effect on PSA dynamics best (based on GOF plots, model convergence and precision of parameter estimates [17]) and parameters could not be reliably estimated using an E_MAX_ model or a sigmoid E_max_ model to describe the direct drug effect. K_D, direct_ was estimated 0.000335 L·day^−1^·GBq^−1^ (40.1% RSE). Addition of a delayed [^177^Lu]Lu-PSMA-I&T effect greatly improved the model fit (dOFV -177). The parameter describing first-order delay to the effect compartment (k_e0_) was estimated 0.00128 h^−1^ (13.1% RSE), while the drug induced delayed effect (k_D, delay_) was estimated 0.0000328 L·day^−1^·MBq^−1^ (17.4% RSE). For IIV on k_G_, a box-cox transformation was applied, since strong deviation from the log-normal distribution was observed and the transformed IIV clearly improved the model fit (dOFV -28.7). An IIV on k_D, delayed_ was added to capture potential different post-treatment effects. IIV on PD parameters was considerable, with 179%, 90.9%, 140% and 87.5% (all CV) on baseline PSA, k_G,_ k_D, direct_ and k_D, delay_, respectively. RUV was best described by a proportional error model.

**Fig. 3:**
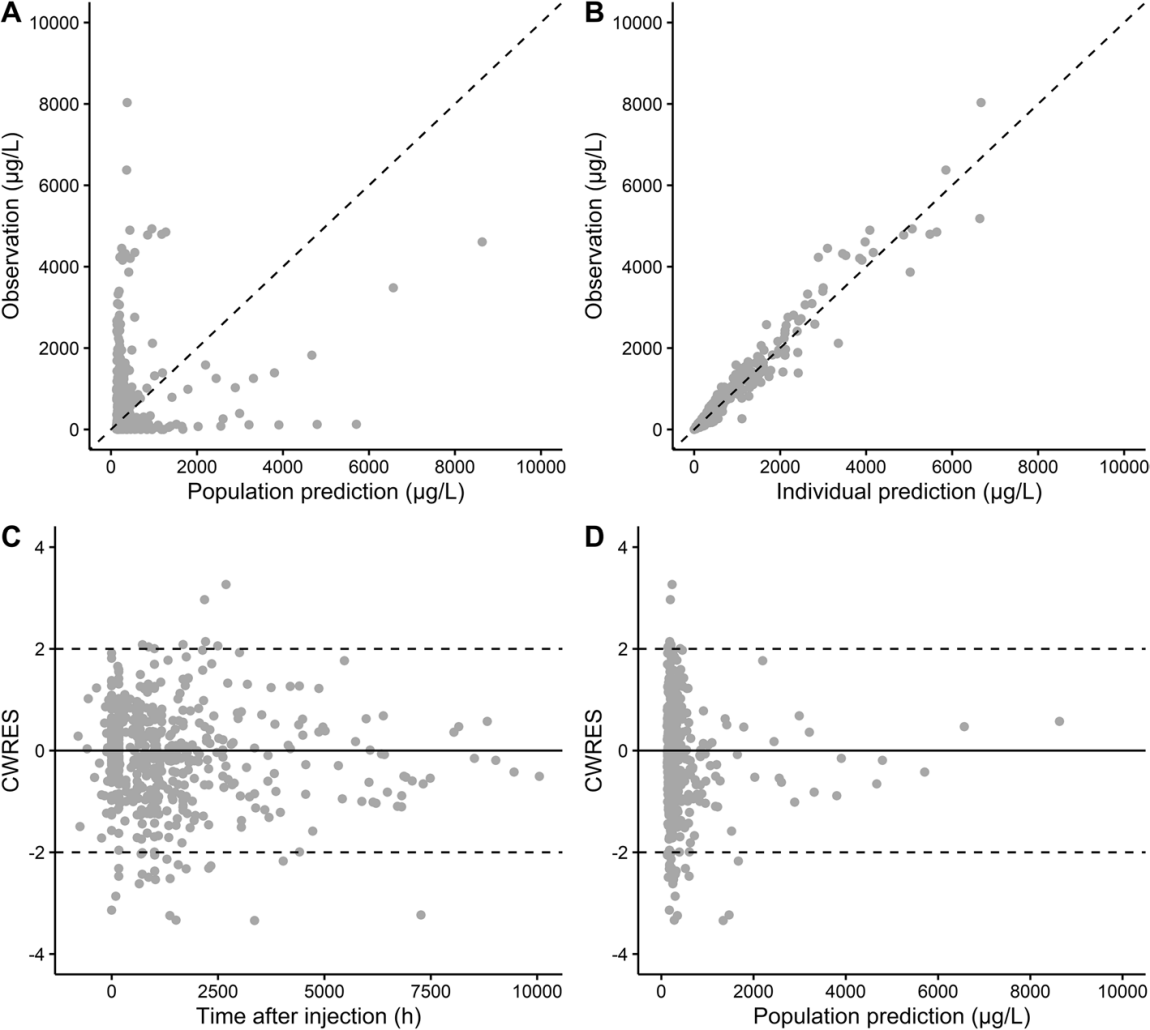
Goodness-of-fit plots of the final population PKPD model for [^177^Lu]Lu-PSMA-I&T, including PSA population predictions (PRED) versus PSA observations (**A**), PSA individual predictions (IPRED) versus PSA observations (**B**), conditional weighted residuals (CWRES) versus time after injection (**C**) and CWRES versus PRED (**D**)

**Fig. 4:**
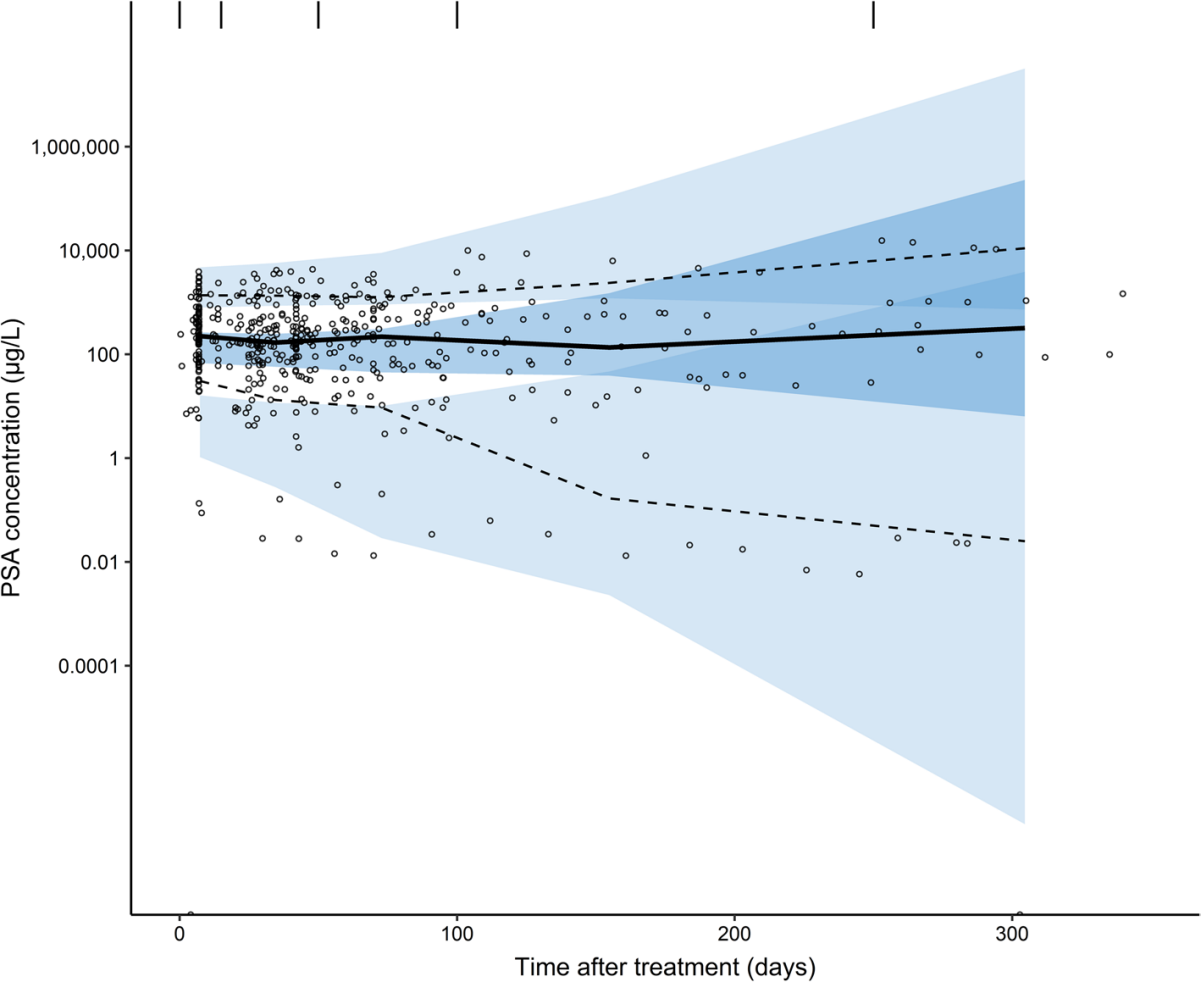
Prediction corrected visual predictive check of the final PKPD model for [^177^Lu]Lu-PSMA-I&T describing PSA dynamics (based on 1000 simulations). Solid lines and dashed lines represent median observed values and 10th and 90th percentiles of observed values, whereas dark and light blue areas represent 95% confidence intervals of the simulated median and 10th and 90th percentiles of simulated values

Based on this final PKPD model, GOF plots (Fig. 3) and the pcVPC (Fig. 4) showed that the model was able to adequately capture PSA dynamics over time. Discrepancies between population predictions and PSA observations were mainly caused by patients that showed extreme high or low baseline PSA values and subsequent PSA concentrations. Still, these observations were captured by the model by including IIV, as is also shown in the pcVPC where simulated variability captured observed variability in PSA concentrations. Plots comparing model predictions with individual PSA observations are provided in Additional file 1: Figure S4. All parameter estimates for the PD model and according parameter precision are shown in Table 3.

**Table 3:** Pharmacodynamic model estimates of the final model for [^177^Lu]Lu-PSMA-I&T

Pharmacodynamic parameters	Estimate (RSE%)	95% CI
*Structural parameters*		
Baseline PSA (µg/L)	140^a^	
Tumor volume on baseline PSA (µg/L)^b^	57.5 (38.9%)	15.5–101.6
PSA growth rate (k_G_) (h^−1^)	0.000408 (14.2%)	0.000286–0.000517
Direct drug-induced effect (k_D, direct_) (L·day^−1^·GBq^−1^)	0.00335 (40.1%)	0.000961–0.006147
Rate constant effect compartment (k_e0_) (h^−1^)	0.00128 (13.1%)	0.00105–0.00171
Delayed drug-induced effect (k_D, delay_) (L·day^−1^·MBq^−1^)	0.0000328 (17.4%)	0.0000235–0.0000450
Box-cox shape parameter	−0.822 (28.3%)	−1.24 to −0.414
*Inter-individual variability*		
Baseline PSA (CV%)	179 (17.4%)	151–210
k_G_ (CV%)	90.9 (32.6%)	64.2–121
k_D, direct_ (CV%)	140 (51.2%)	61.0–206
k_D, delay_ (CV%)	87.5 (26.1%)	66.1–109
*Residual unexplained variability*		
Proportional error (CV%)	29.3 (9.2%)	27.0–32.2

95% CI and RSE values were obtained from the SIR

*CI* confidence interval, *CV%* coefficient of variation,* PSA* prostate-specific antigen, *RSE* relative standard error, *SIR* sampling importance resampling

^a^Fixed parameter

^b^Added using a linear covariate function: $P_{cov} = P_{pop} + \left( { \theta_{cov} *\left( {\frac{COV}{{COV_{median} }}} \right)} \right)$

### Simulations

**Fig. 5:**
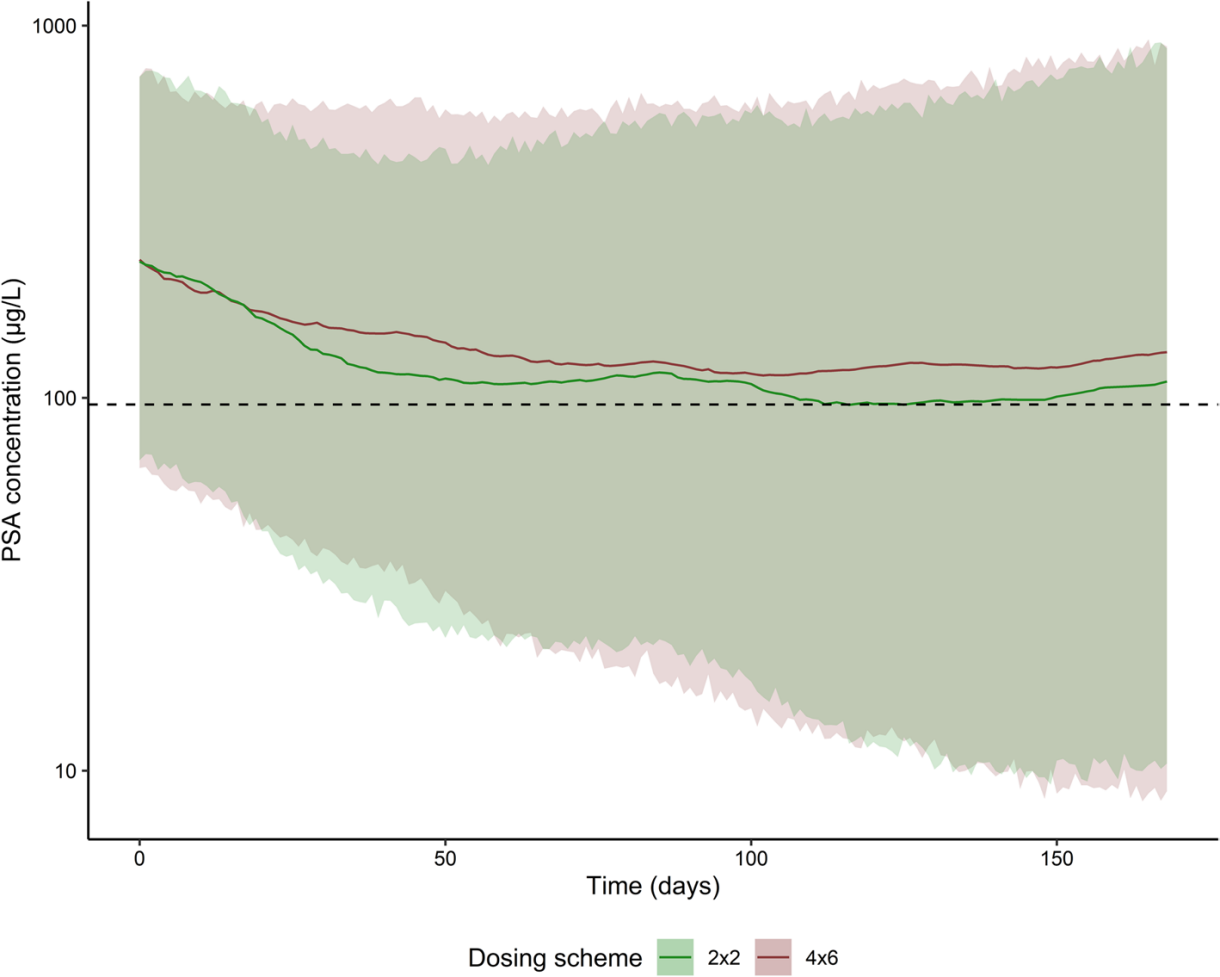
Simulated PSA dynamics based on the final population PKPD model (median simulation with 50% prediction interval, n = 2000) after treatment with [^177^Lu]Lu-PSMA-I&T with two different dosing schemes: two cycles of 7.4 GBq with a two-week interval (series repeated after twelve weeks) (‘2 × 2’) and four cycles of 7.4 GBq with a six-week interval (‘4 × 6’). Simulated results implied a slightly more prominent decrease in PSA (especially after the second cycle) for the ‘2 × 2 - repeated after twelve weeks’ scheme

Simulations based on the final model showed only slight differences in PSA dynamics between both dosing schedules (‘4 × 6’ vs ‘2 × 2 − repeated after twelve weeks’) (see Fig. 5). Stable disease (i.e. no increase in PSA) was predicted for 54.8% vs 56.4% of all patients after the ‘4 × 6’ and ‘2 × 2 − repeated after twelve weeks’ dosing schedule, respectively. Response to therapy with a ≥ 50% decrease in PSA was predicted for 42.4% and 44.7% of all patients for the ‘4 × 6’ and ‘2 × 2—repeated after twelve weeks’ dosing schedule, respectively. A simulated difference in PSA concentrations was visually observed between both dosing regimens during the simulated follow-up period, where the ‘2 × 2 − repeated after twelve weeks’ scheme showed a more prominent decrease in PSA (shown in Fig. 5).

### Exposure thresholds

**Fig. 6:**
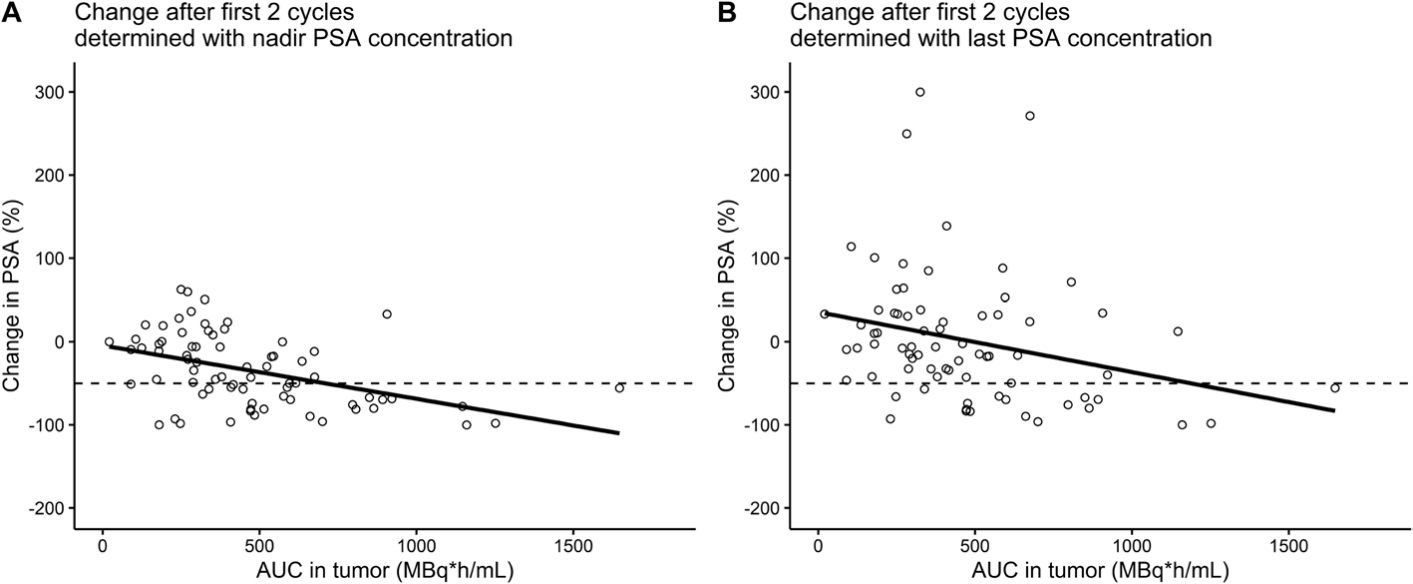
Relationship between the observed change in PSA and the individually estimated cumulative area under the curve (AUC) in tumors (i.e. time-integrated activity) after the first two treatment cycles, where the change in PSA was determined based on the nadir value (**A**) and the last measured value (**B**) compared to the baseline PSA value

Linear regression analyses showed an evident relationship between tumor exposure (AUC) and change in PSA (for both nadir PSA as endpoint measure and the last measured PSA concentration as endpoint measure) (see Fig. 6). Treatment response (≥ 50% decrease in PSA) was calculated to occur with a threshold AUC of 709.5 MBq·h/mL in case the nadir PSA value was the outcome measure, while 1188 MBq·h/mL AUC was required for obtaining a ≥ 50% decrease in PSA at the last measurement after the first two cycles.

## Discussion

A population PKPD model was developed for [^177^Lu]Lu-PSMA-I&T based on clinical imaging data of 76 patients with mCRPC. The PKPD model showed adequate description of PSA changes over time during and following treatment with [^177^Lu]Lu-PSMA-I&T. Radioactivity concentrations in the tumor compartment proved informative to describe PSA dynamics as a measure of treatment response, indicating the presence of an exposure–response relation. This was the first study to quantify PSA response based on PK of [^177^Lu]Lu-PSMA-I&T using a population modelling approach. Further elaboration regarding the identified cycle effect (and random cycle-to-cycle variability), the quantified direct and delayed effects of [^177^Lu]Lu-PSMA-I&T tumor exposure on PSA dynamics and the simulated response results for both dosing regimens are provided below.

### PK model (structure)

The PK model structure resembles a lumped physiologically based pharmacokinetic (PBPK) model including only the organs of interest (i.e. a minimal PBPK model [45]). Lumped compartments (without distinction between subcompartments) were used, because nuclear imaging data do not allow intra-compartment distinction of the location of the radioactivity and assumptions are always required to make such distinctions (e.g. using PBPK models). As a result, not all system-specific parameters, drug-specific parameters and physiological processes as required for a full PBPK were necessary and a simpler description of drug-transfer could be used. In addition, parameters and their variability were estimated (i.e. top-down approach) rather than defined beforehand based on drug and system specific prior knowledge (i.e. bottom-up approach). All parameters could be fitted since data observations for most (lumped) compartments were available, resulting in an identifiable model. This approach enabled straightforward individual Bayesian estimation of parameter estimates and description of uptake in all compartments, which will be relevant in future individual predictions. More information on differences between population PK and PBPK modelling approaches can be found in literature [46].

Renal clearance was estimated 2.61 L/h, which is comparable to the reported clearance for [^177^Lu]Lu-PSMA-617 of 2.04 L/h [47]. Comparison of rate constants for drug transfer between different compartments is hampered by absolute radioactivity amounts being relatively small in tumors compared to organs (due to volume differences between these compartments). However, these parameter differences showed that maximum concentrations are achieved slower in tumors. The volume of distribution (V1) of 10.3 L reflects that, apart from receptor binding, the drug is hydrophilic and remains mainly in the blood. Kratochwil et al. reported an initial volume of distribution of 22 L for [^177^Lu]Lu-PSMA-617 [48], which approximated extracellular body water. Taking into account our additional description of separate compartments, these results are quite comparable. Estimated half-lifes for organs-at-risk and tumors were approximately 11 h and 46 h, respectively. For tumors, this is comparable to previous reported half-life of 51 h, while for organs our estimated half-life is clearly lower than the reported 33 h [49]. This might be due to the different PSMA-ligand.

A limitation of our PK model is the number of available data points per patient. Unfortunately, only two or three scans per cycle were available for PK model development, which is due to the retrospective nature of our study and thus could not be avoided. Also, in clinical practice, three imaging moments is considered the maximum, as post-treatment imaging has a major impact on the wellbeing of patients and places a burden on department logistics.

### Cycle-to-cycle effects

The cycle-to-cycle variability of 37.8% (CV) on the tumor uptake rate (k_14_) was hypothesized to play a part in [^177^Lu]Lu-PSMA-I&T PK based on previously reported results for [^177^Lu]Lu-PSMA-617, where a somewhat higher CV was found of 43.5% [24]. Though differences are small, these might be caused by different treatment intervals (two-week instead of six-week interval) for most patients. Excretion rates of these slightly different PSMA-ligands were comparable (0.253 h^−1^ for PSMA-I&T vs 0.288 h^−1^ for PSMA-617) [24]. Covariate analysis during population PK model development showed that tumor uptake rates evidently decrease in subsequent treatment cycles to 73.1%, 49.8% and 43.6% in cycle 2, 3 and 4–7, respectively, compared to cycle 1. Recently, first clinical evidence for this cycle effect was published in a small population treated with [^177^Lu]Lu-PSMA-I&T (n = 5–15 patients per cycle) [50]. The average tumor absorbed dose decreased in this study from 3.5 Gy/GBq in the first cycle to 3.3 Gy/GBq (94%), 2.7 Gy/GBq (77%) and 2.4 Gy/GBq (68%) in the second to fourth cycle. Reduced tumor uptake in later cycles could be due to tumor cell kill and/or a decrease in PSMA receptor expression, caused by radiation damage to all previously targeted cells and/or receptors. Also, tumor vascularization might be harmed due to radiation effects, since some evidence for this phenomenon was recently provided [51]. A similar cycle-effect was also tested on uptake in salivary glands (being an organ-at-risk for toxicity), where a slight decrease was observed in cycle two (84.6%) compared to cycle one, whereas this decreased uptake diminished in cycle three (98.1%). This indicated that salivary gland tissues are not capable to recover from radiation induced damage in two weeks, while after approximately twelve weeks the cells seem restored and/or compensated uptake (e.g. by increased expression or perfusion), so that accumulation was similar to start of treatment. The limited capabilities for tissue regeneration and remodeling after radiation induced damage for the tumor (microenvironment) compared to normal tissues is one of the cornerstones of radiation-based therapies. The fact that a gradual decrease in accumulation over cycles is present for tumors, but to a far lesser extent in normal organs, is highly relevant and underlines the need for more personalized dosing schemes as opposed to fixed dosing regimens (e.g. by increasing the injected radioactivity in initial cycles).

### Modelling PSA dynamics

Since this is the first PKPD model for [^177^Lu]Lu-PSMA-I&T, evaluation of estimated PD parameters is challenging. PSA growth seemed captured accurately, since k_G_ of 0.000408 h^−1^ is comparable to a previous estimated PSA growth rate of 0.000366 h^−1^ in castration resistant PCa patients [37]. Precision of the parameters related to the PD part of the model was less adequate. Hence, the developed model could be improved in future research. First, current data in tumors only represented segmented tumors. Since many patients have extensive metastases and the included tumor lesions do not represent overall tumor uptake, this might not fully reflect PSA changes. Secondly, the first ten patients treated in our hospital were selected based on extreme late-stage disease. Patients currently treated with [^177^Lu]Lu-PSMA-I&T in our hospital are, generally speaking, in a better clinical condition and these two groups (i.e. ‘4 × 6’ and ‘2 × 2 − repeated after twelve weeks’) in our included patients might not reflect one population of patients. Still, data of all patients was included for PK model development, to include all available information on both dosing regimens. Thirdly, additional PSA observations might improve the model fit even further. Lastly, an additional covariate analysis in a larger patient population will be useful, so that IIV might be further explained and PSA dynamics is even better captured.

The simulated response rates (patients showing a ≥ 50% decrease in PSA) after [^177^Lu]Lu-PSMA therapy based on our PKPD model was comparable to published large population data, with 42.4% and 44.7% of all patients for the ‘4 × 6’ and ‘2 × 2 − repeated after twelve weeks’ dosing schedule, respectively, versus 46.0% and 52.0% in the VISION trial and REALITY study, respectively [7, 22]. Our simulations showed somewhat comparable PSA responses 24 weeks after start of treatment for both dosing regimens, though the ‘2 × 2 − repeated after twelve weeks’ dosing scheme showed an evident decrease direct after the second cycle. By comparing responses at 24 weeks after start of treatment, it should be noted that this was ten weeks after the last treatment cycle for the ‘2 × 2 − repeated after twelve weeks’ group, whereas six weeks after the last treatment cycle for the ‘4 × 6’ group. In addition, the eventual response effect might be underestimated for the ‘2 × 2—repeated after twelve weeks’ group, since patients not responding to therapy will not receive a second therapy series. Possibly, administering the second treatment series earlier after the first treatment series might result in a larger decrease in PSA. Also, the patient selection based on the initial treatment cycle should be improved in future research, to avoid treating patients that are unlikely to respond to therapy. Our simulation results implied that the ‘2 × 2 − repeated after twelve weeks’ would at least result in similar response rates compared to the ‘4 × 6’ scheme studied in the VISION-trial [7], but might even lead to better response rates in case of further optimization.

The established tumor threshold AUCs are a first step towards this patient selection, since a clear relationship between tumor AUC and therapy outcome is shown. However, the linear relationship comes with uncertainty and might not represent the optimal model, thus further evaluation is required based on additional data. Also, ideally, other response measures should be used to assess threshold AUCs and these need to be compared to these established thresholds. These potential improvements, together with the already comparable simulated response rates, make the ‘2 × 2 − repeated after twelve weeks’ dosing regimen an interesting scheme for radioligand therapy.

### Direct and delayed effects of radiation

Evaluation of the PD model showed an adequate description of PSA dynamics for the majority of patients by including a direct and delayed effect of [^177^Lu]Lu-PSMA-I&T. Regarding the identified direct and delayed effects, interpretation of results needs further elaboration. The direct effect showed a rather low k_D, direct_. Therefore, the direct effect of this radioligand therapy is limited for the majority of patients and the delayed treatment effect clearly contributed to the overall response. The existence of delayed effect was expected, since radiation damage is known to be caused by both direct and indirect action of radiation on the targeted cells [52]. Furthermore, it is of interest to relate our results to observations from external beam radiation therapy and brachytherapy, where cell survival is often modelled using linear-quadratic models [53, 54]. Using these models, the radiation effect on cells is assessed with alpha and beta parameters (a linear and quadratic component, respectively), which both reflect the radiosensitivity of cells (i.e. higher values indicate more sensitive cells) [55]. The alpha/beta ratio indicates the fractionation sensitivity of cells, where generally speaking, the higher the ratio, the more linear the cell survival curve and thus the less sensitive the cells are to fraction. With low alpha/beta ratios, the linear component is less important and this represents late responding tissue with a high sensitivity for fraction sizes [53, 55–57]. PCa showed a low alpha/beta ratio of about 1.5–3 Gy [56–60], which indicated late responding cells and may be in line with our findings of a delayed effect of the radionuclide treatment [56, 57, 61]. In addition, considering repair of PCa cells [57], a new dose is ideally administered close to the previous administration, to limit the possibility of tumor cell repair while simultaneously giving the healthy tissue time to do some repair. This is accomplished by using a ‘2 × 2’ dosing scheme, where the second cycle of [^177^Lu]Lu-PSMA-I&T is administered almost directly after wash-out of the tumor exposure from the previous cycle. This can be compared with radiotherapy models where cell repair is taken into account, such as brachytherapy [62]. Still, increasing injected radioactivity doses might increase efficacy due to the delayed effect on PSA, which is in line with achieving higher tumor doses per fraction as proposed in hypofractionated schemes for low alpha/beta ratio PCa tissue.

The included delayed effect was linearly concentration-dependent. Mechanistic explanations of a delayed treatment effect of [^177^Lu]Lu-PSMA-I&T could be changes in the tumor microenvironment, such as the before mentioned alteration in vascularization [51]. A radiation-induced bystander response could also explain the identified delayed effect, which is a phenomenon that explains how non-irradiated cells could show damage similar to ionizing radiation as a result from neighbor cells being irradiated [63, 64]. Though exact mechanisms of the radiation-induced bystander effect are very complex, the interaction of irradiated cells with the immune system is likely to play an important role [65, 66]. A delayed effect on PSA levels is also described after external beam radiation therapy, since the average time to nadir PSA after radiotherapy is approximately fifteen months [67]. It is important to mention that this delay in PSA effect does not per se reflect a similar delayed effect on overall tumor treatment response. The estimated delay is probably also partly related to PSA being our PD measure, because changes in PSA are likely to occur somewhat after treatment [67, 68]. This could be partly due to occurrence of a PSA-flare phenomenon after start of treatment, which was previously hypothesized before for radioligand therapy with [^177^Lu]Lu-PSMA-617 [68, 69]. In that case, PSA does not immediately decrease after start of treatment due to release of PSA out of responding tumor cells. Therefore, caution is also warranted with interpretation of direct effect results, since the identified direct effect refers to a direct effect on PSA response (and not per se overall treatment response).

### Future applications

Using a PKPD modelling approach, an evident exposure–response relationship was identified for [^177^Lu]Lu-PSMA-I&T, where tumor accumulation was related to direct and delayed effects on PSA dynamics. Eventually, this population model could form the basis to individually predict tumor exposure and treatment response using Bayesian forecasting (similar as applied in a therapeutic drug monitoring approach [70]). Baseline PSA levels combined with observed uptake in tumors and all prior known patient characteristics (i.e. covariates in the model) can be used to estimate individual Bayesian estimates (based on previous established population parameters, IIV, IOV and RUV). These Bayesian parameter estimates can then be used to predict individual PSA changes over time. In addition, since the debate on how to evaluate treatment response is ongoing, future PKPD models should also include RECIST criteria or tumor growth as PD measures [71]. The approach of predicting individual response to therapy could guide individualized dosing or selection of patients for radioligand therapies.

## Conclusions

Our population PK model accurately described observed radioactivity in salivary glands, kidneys and tumors after [^177^Lu]Lu-PSMA-I&T treatment in patients with mCRPC. A declining tumor uptake over cycles was revealed and tumor uptake was estimated to decrease to 73%, 50% and 44% in cycle 2, 3 and 4–7, respectively, compared to cycle 1. Higher tumor accumulation was related to better PSA response, explained by both a direct and delayed effect of [^177^Lu]Lu-PSMA-I&T therapy. The final PKPD model adequately captured individual PSA observations and identified population response rates and tumor AUC thresholds. Using such a PKPD modelling approach in future research could help to individually predict treatment outcome and, thus, identify patients in whom radioligand therapy is likely to fail.

### Supplementary Information

**Additional file 1.** Individual model fit results.

## Data Availability

The datasets used for the current study are available from the corresponding author on reasonable request.
