# Supplementary material for: Quantification of biochemical PSA dynamics after radioligand therapy with [177Lu]Lu-PSMA-I&T using a population pharmacokinetic/pharmacodynamic model
Source: EJNMMI Phys. 2024 Apr 24;11:39. doi: 10.1186/s40658-024-00642-2 (PMC11043318; doi:10.1186/s40658-024-00642-2)
Supplement: Supplementary file 1 — Additional file 1. Individual model fit results. [file 40658_2024_642_MOESM1_ESM.docx]

**Supplementary Material**

**Quantification of Biochemical PSA Dynamics after Radioligand Therapy with [^177^Lu]Lu-PSMA-I&T using a Population Pharmacokinetic/Pharmacodynamic Model**

Hinke Siebinga ^1,2^, Berlinda J. de Wit-van der Veen ^2^, Daphne M.V. de Vries-Huizing ^2^, Wouter V. Vogel ^2,3^, Jeroen J.M.A. Hendrikx ^1,2^, Alwin D.R. Huitema ^1,4,5^

1 Department of Pharmacy & Pharmacology, The Netherlands Cancer Institute: Antoni van Leeuwenhoek, Amsterdam, The Netherlands

2 Department of Nuclear Medicine, The Netherlands Cancer Institute: Antoni van Leeuwenhoek, Amsterdam, The Netherlands

3 Department of Radiation Oncology, The Netherlands Cancer Institute: Antoni van Leeuwenhoek, Amsterdam, The Netherlands

4 Department of Clinical Pharmacy, University Medical Center Utrecht, Utrecht University, Utrecht, The Netherlands

5 Department of Pharmacology, Princess Máxima Center for Pediatric Oncology, Utrecht, The Netherlands

**Corresponding author:** h.siebinga@nki.nl, The Netherlands Cancer Institute, Plesmanlaan 121, 1066 CX Amsterdam

**
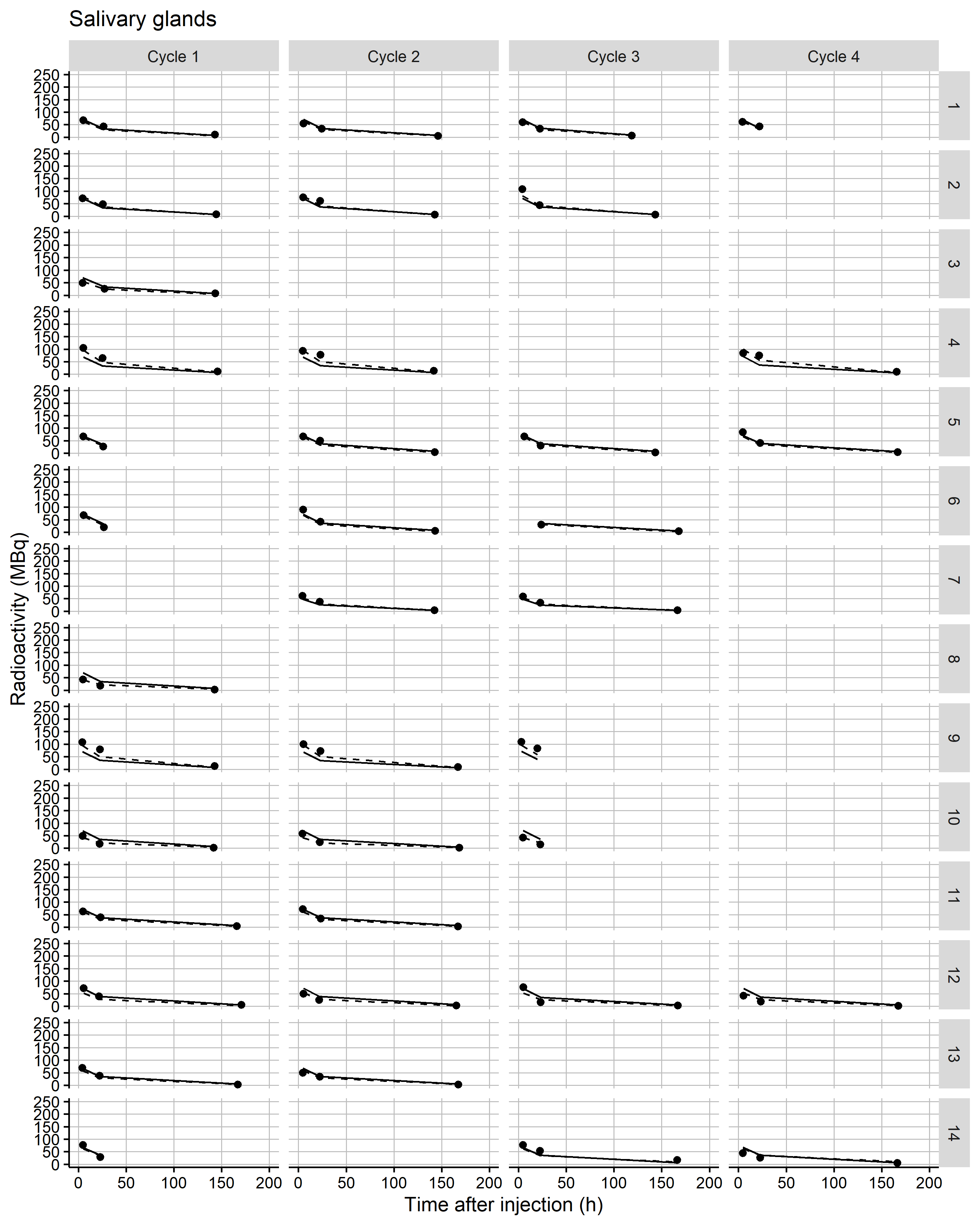

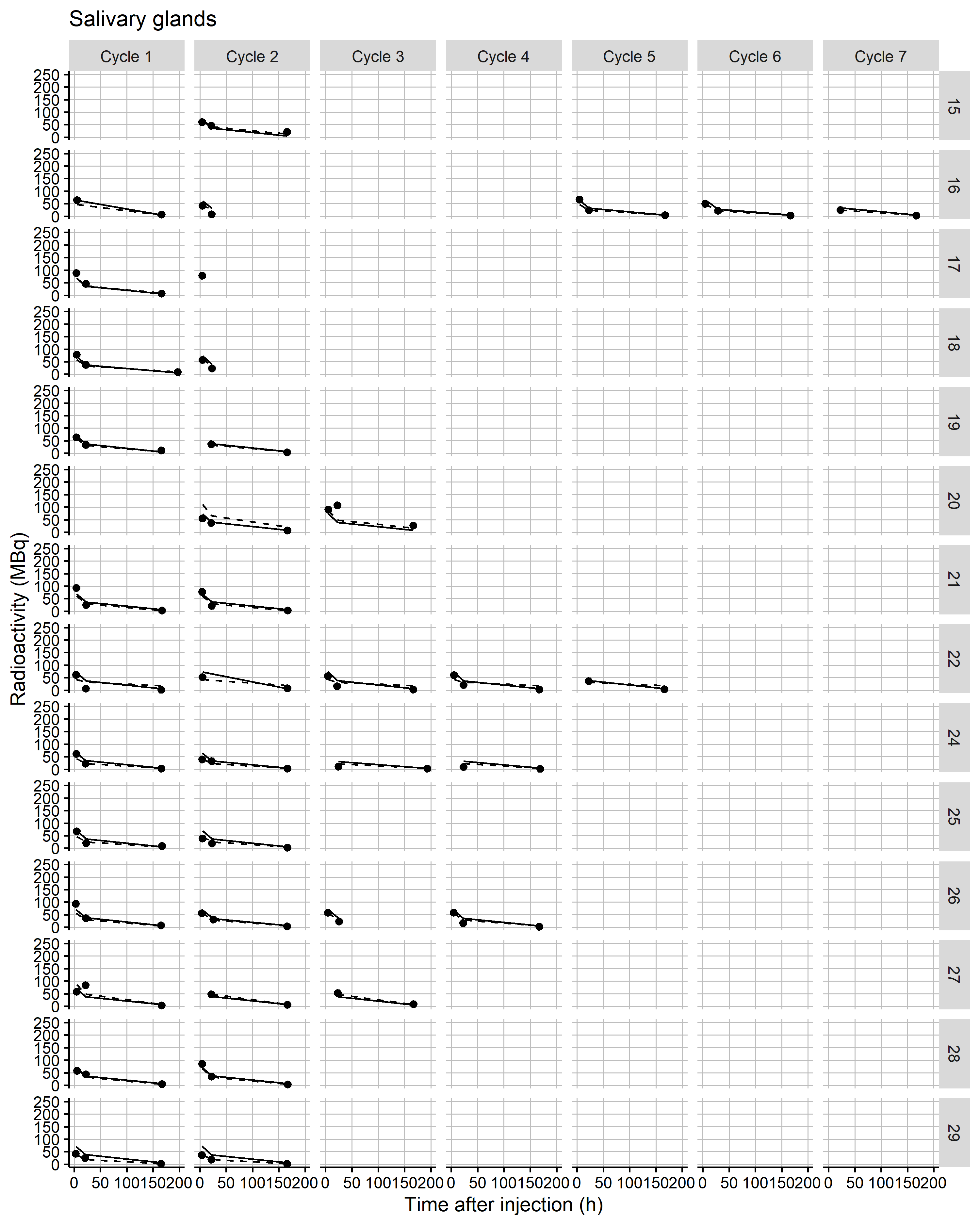

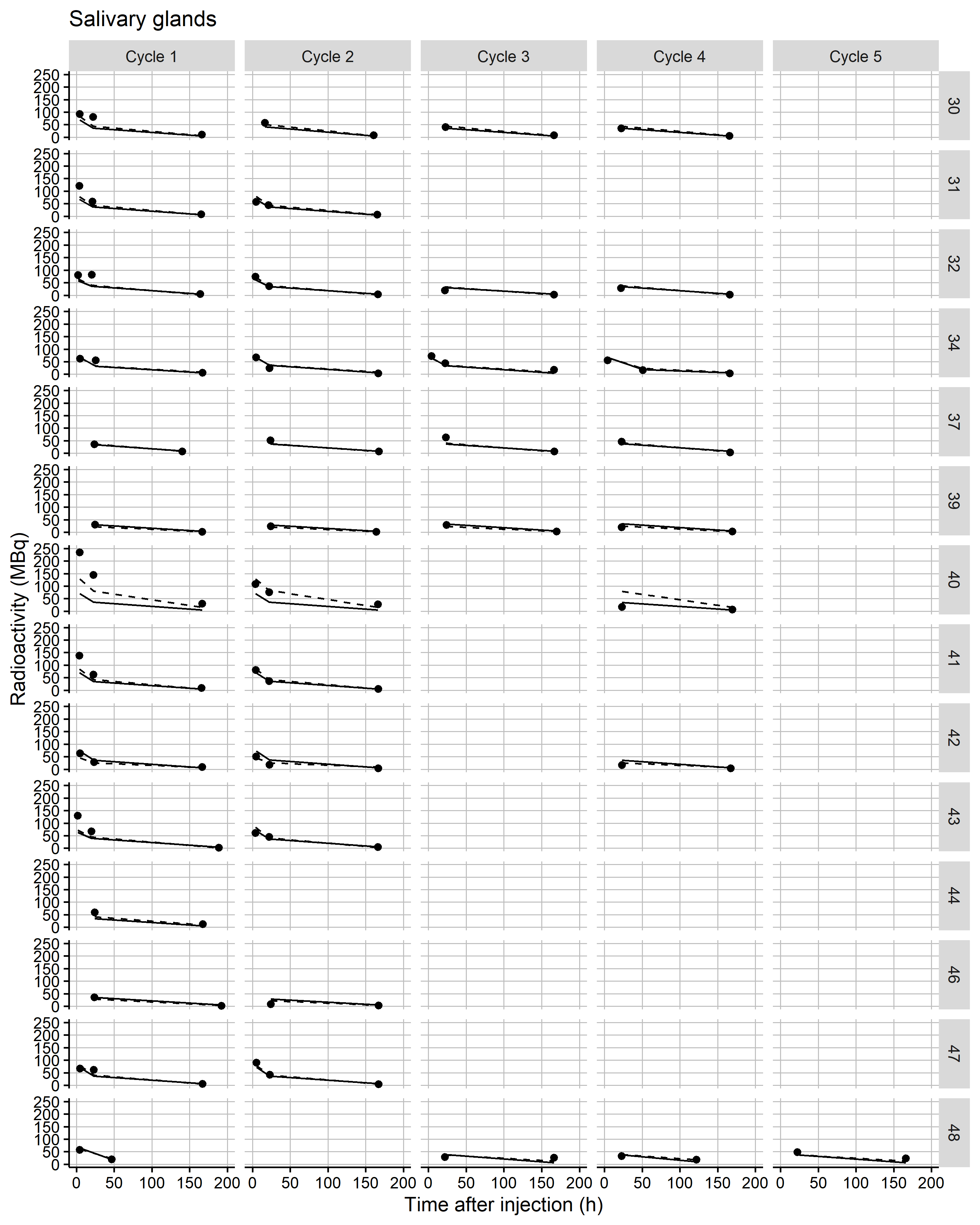

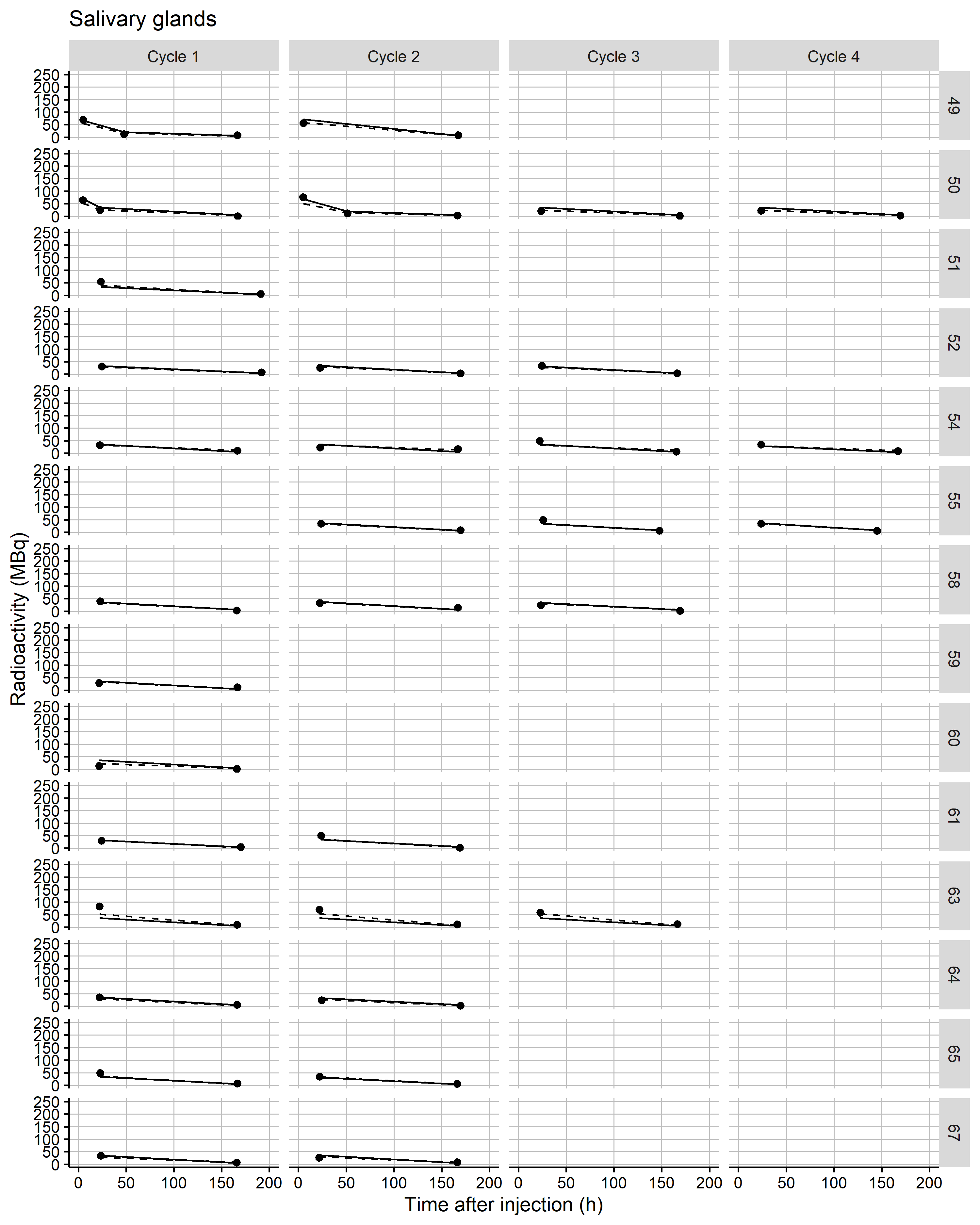

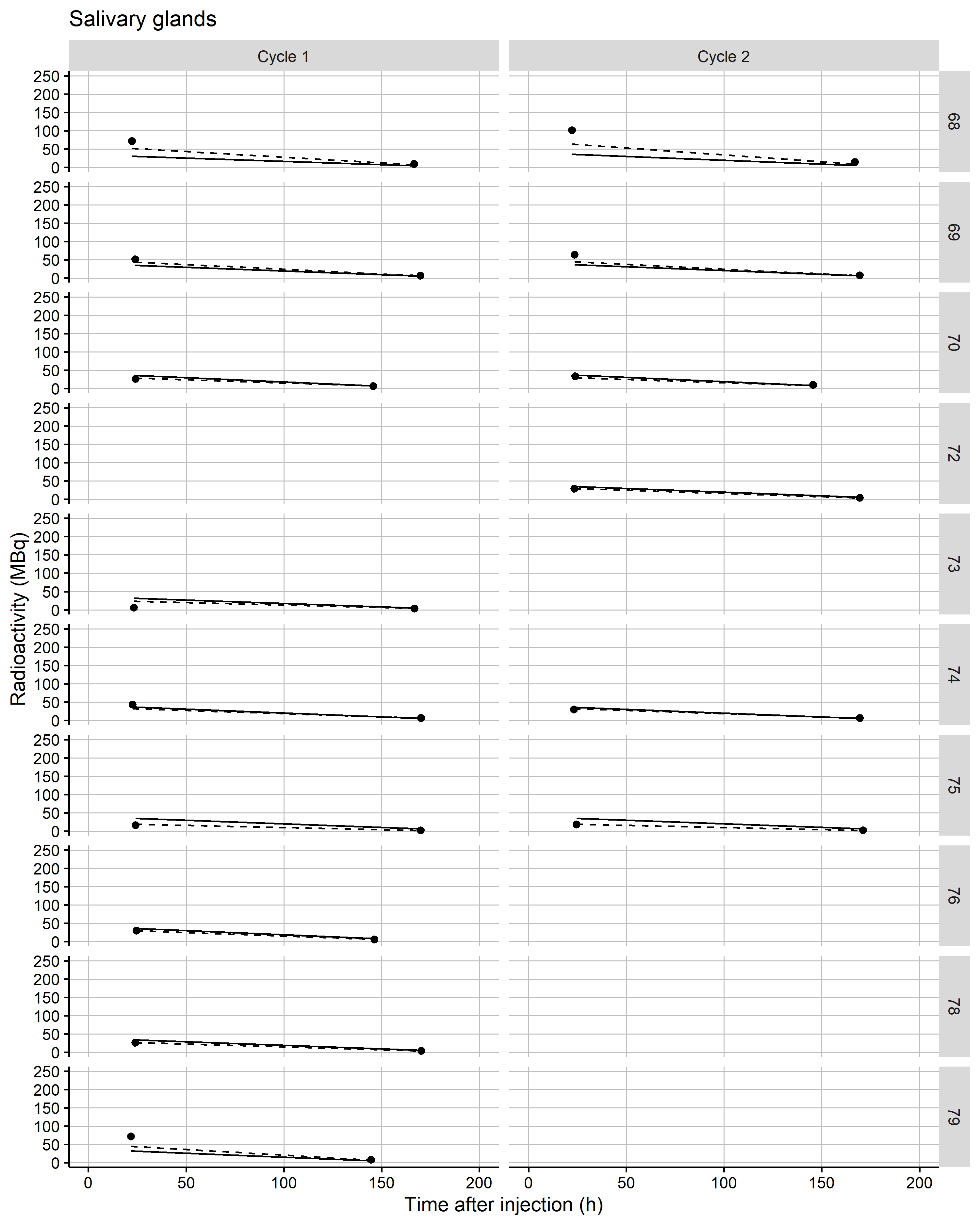
**

**Supplemental Figure 1** – Individual [^177^Lu]Lu-PSMA-I&T concentration over time plots for salivary glands, where individual (dashed lines) and population (solid lines) predictions based on the final PKPD model as well as observed data (dots) are shown (per cycle).

**
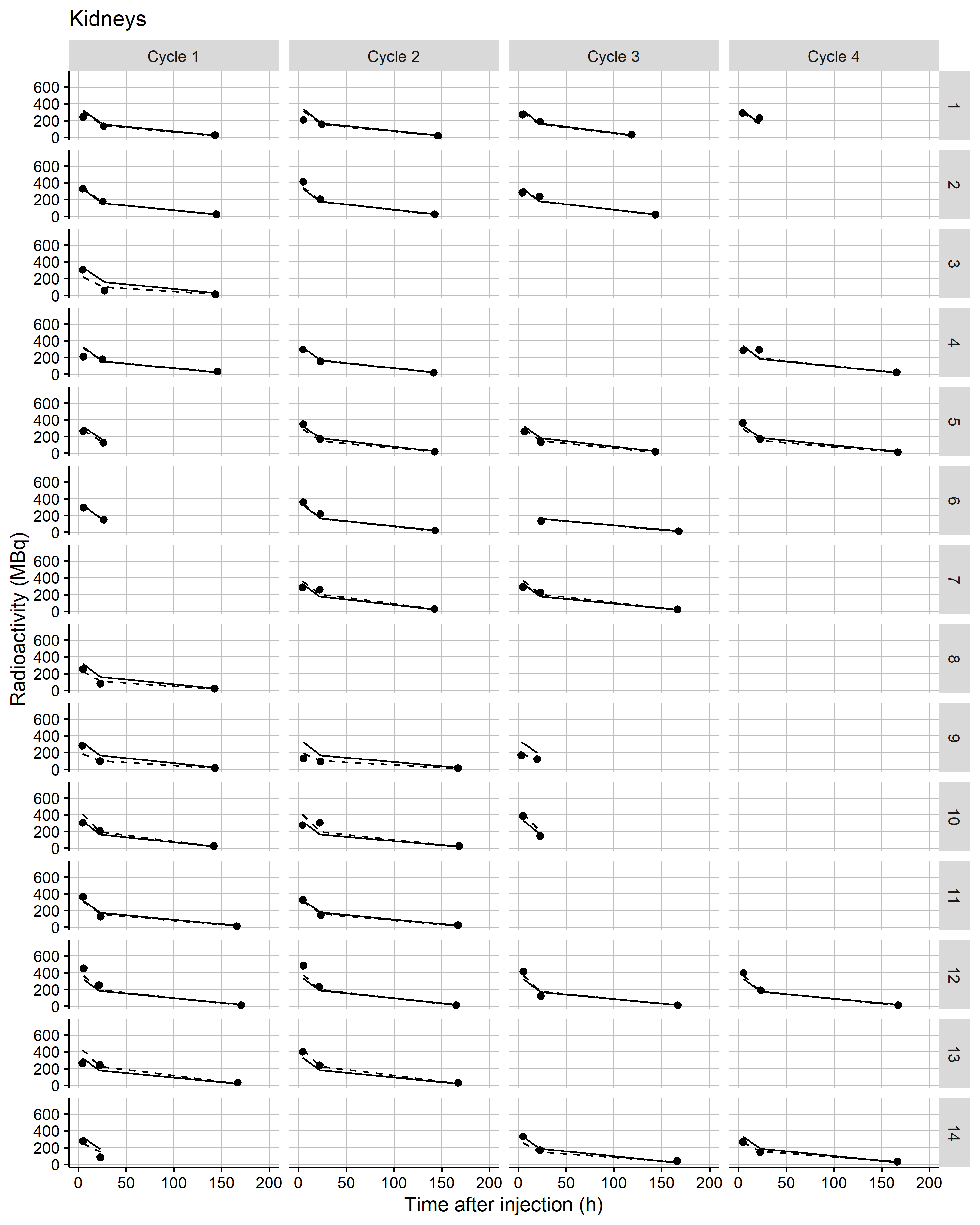

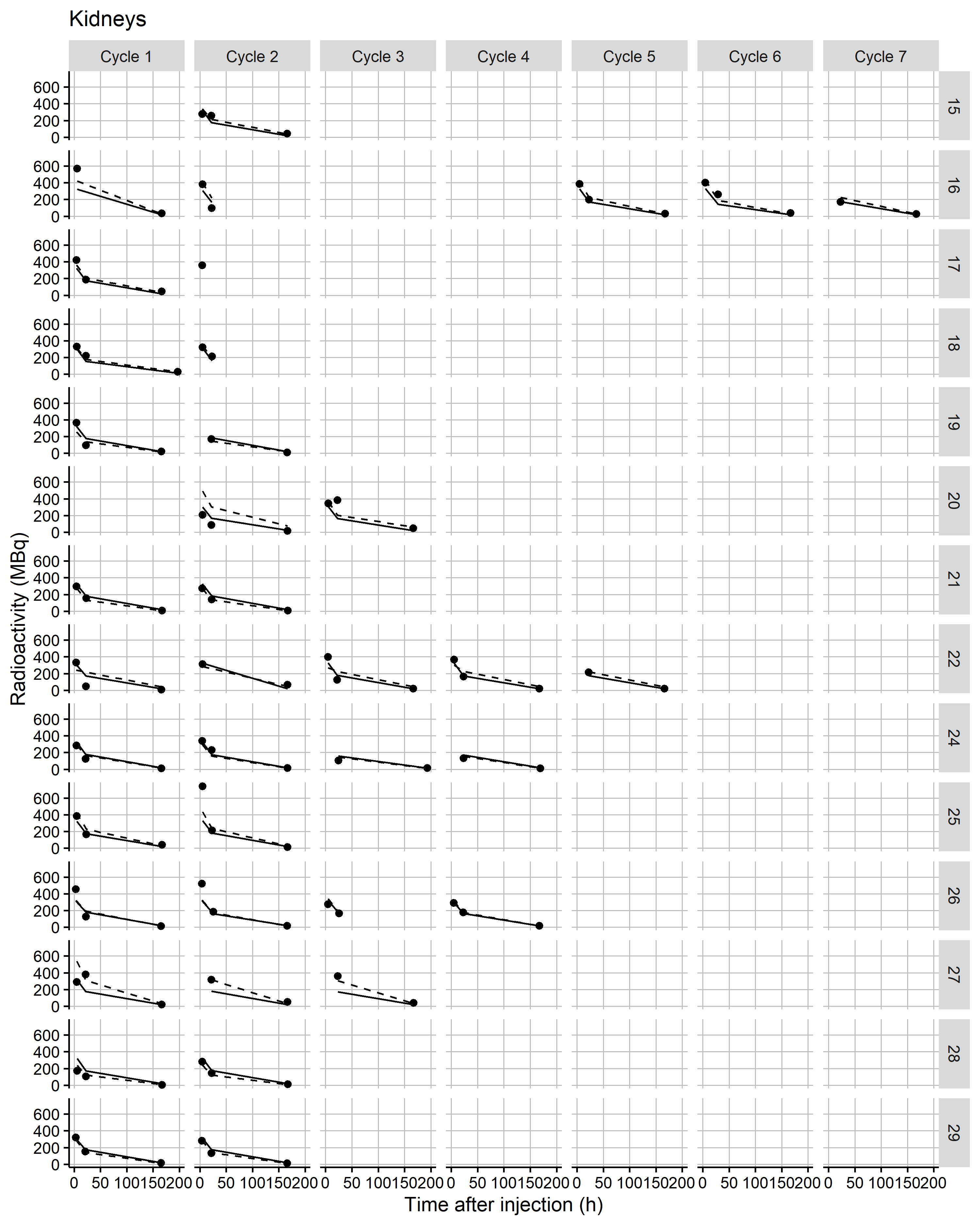

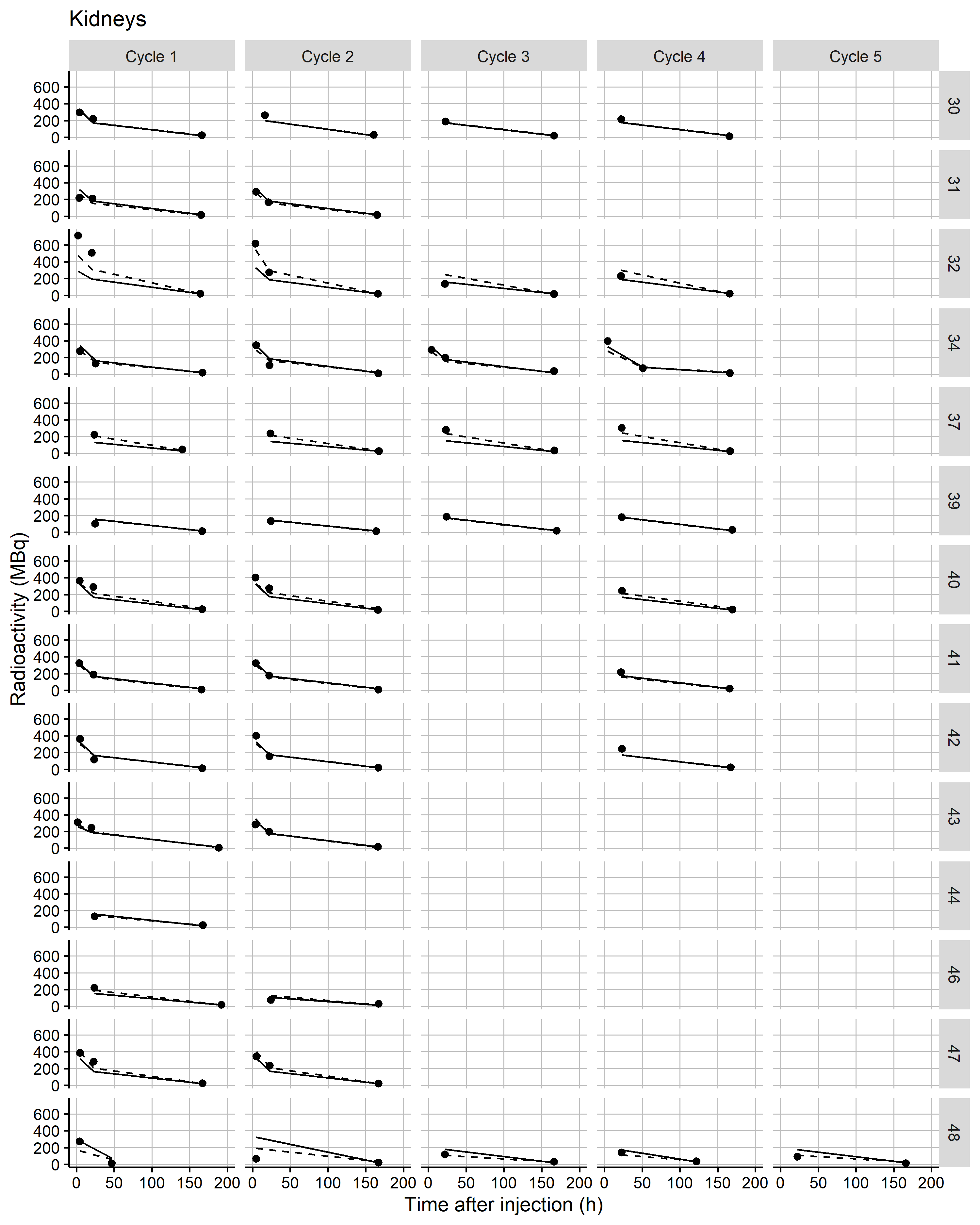

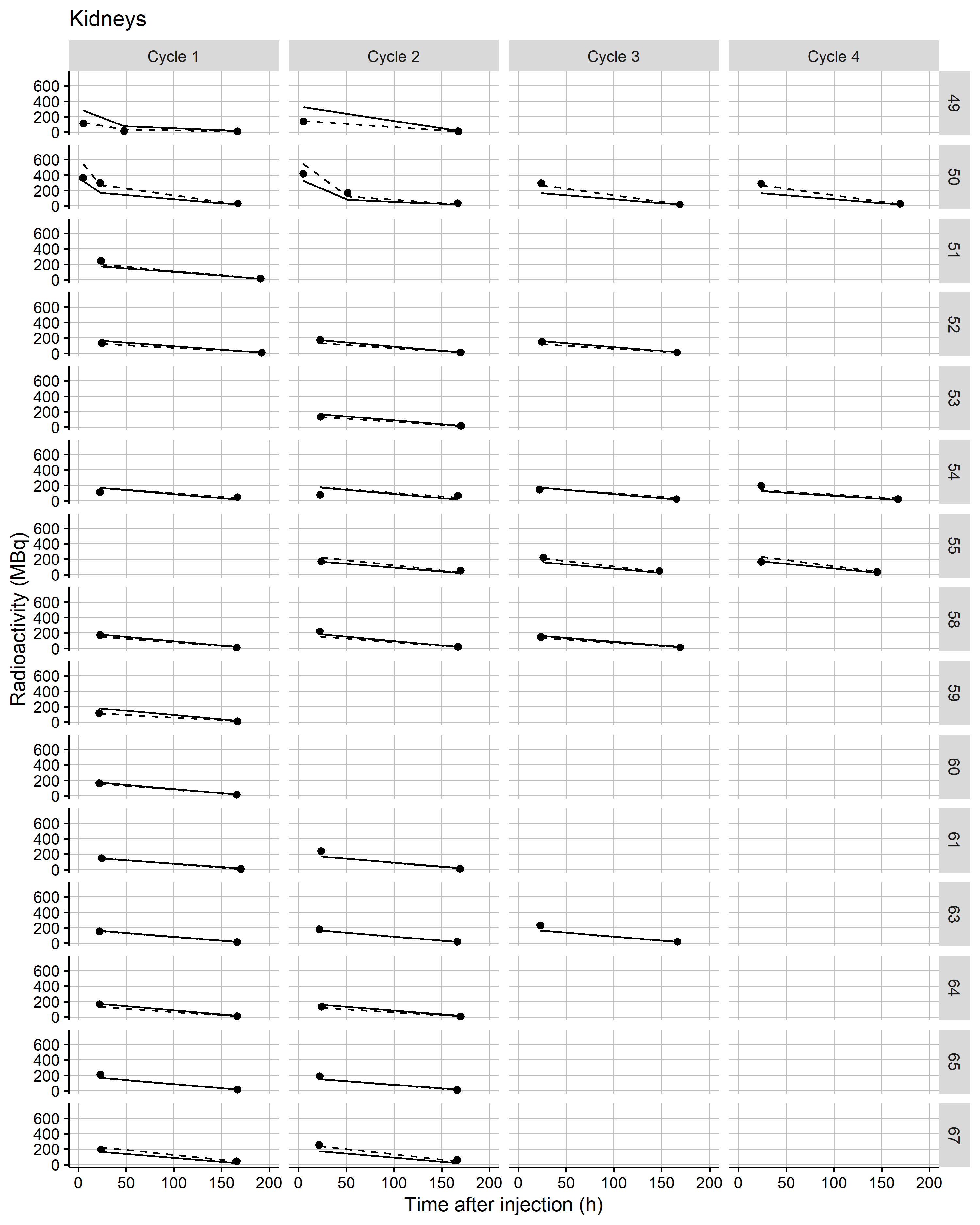

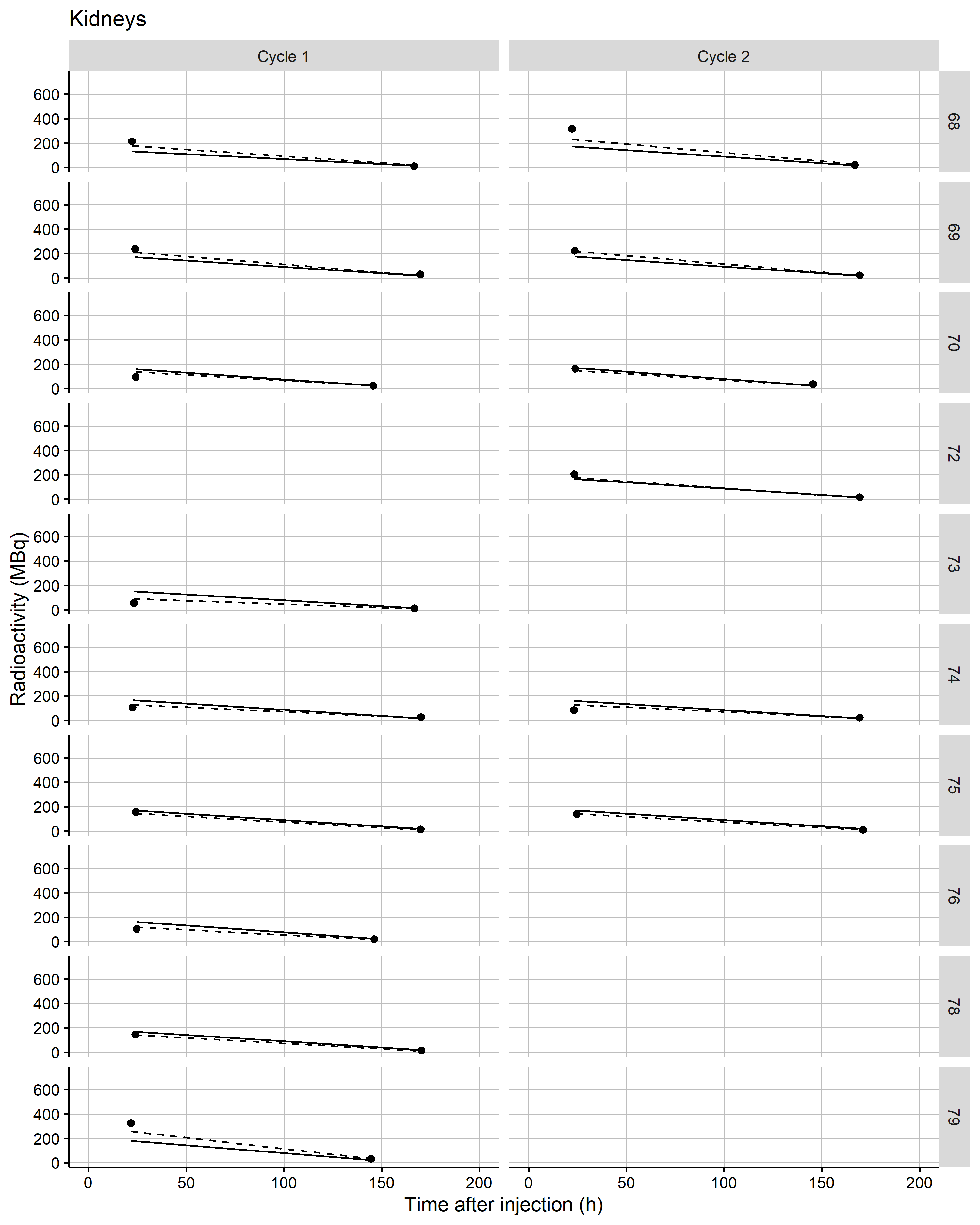
**

**Supplemental Figure 2** – Individual [^177^Lu]Lu-PSMA-I&T concentration over time plots for kidneys, where individual (dashed lines) and population (solid lines) predictions based on the final PKPD model as well as observed data (dots) are shown (per cycle).

**
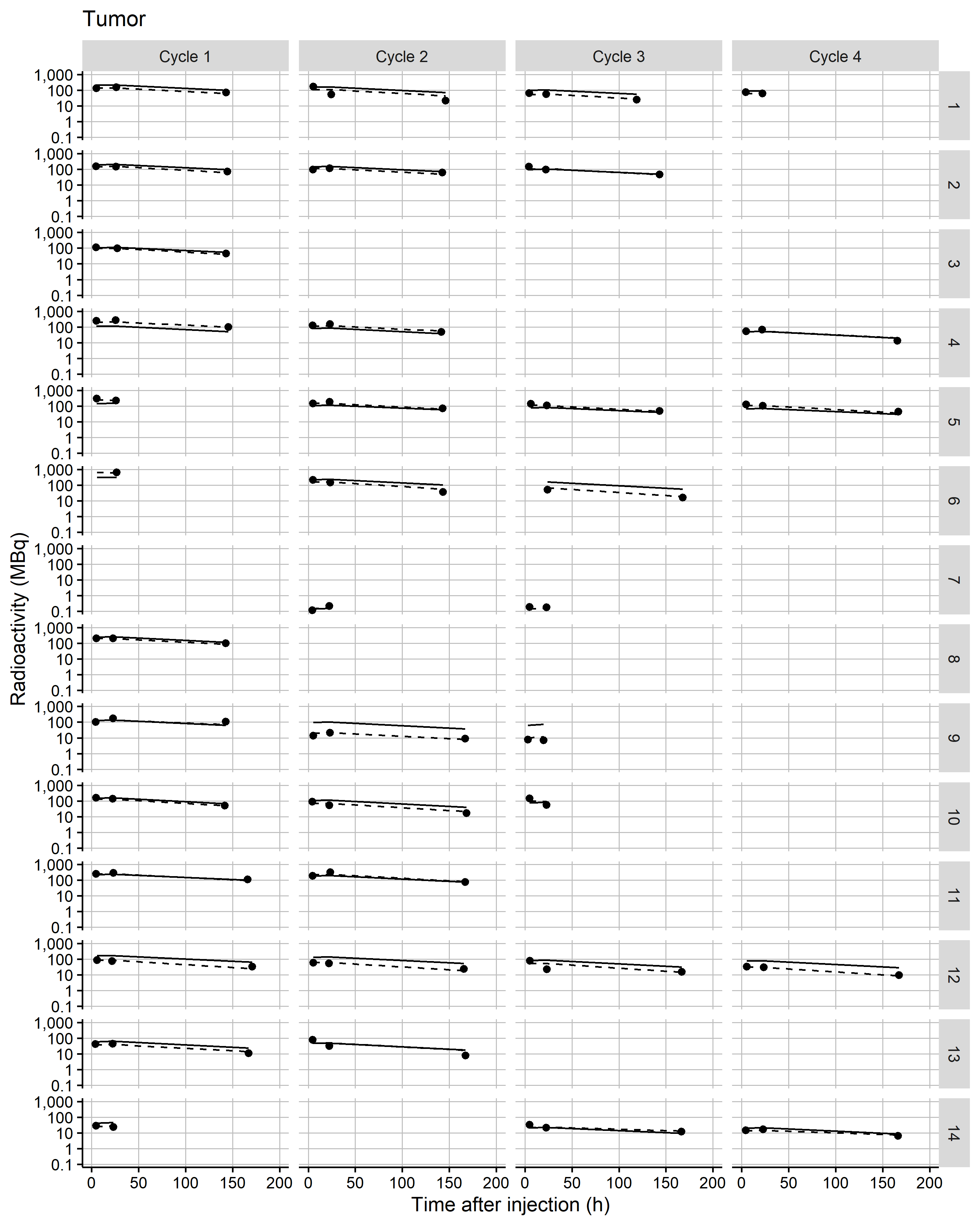

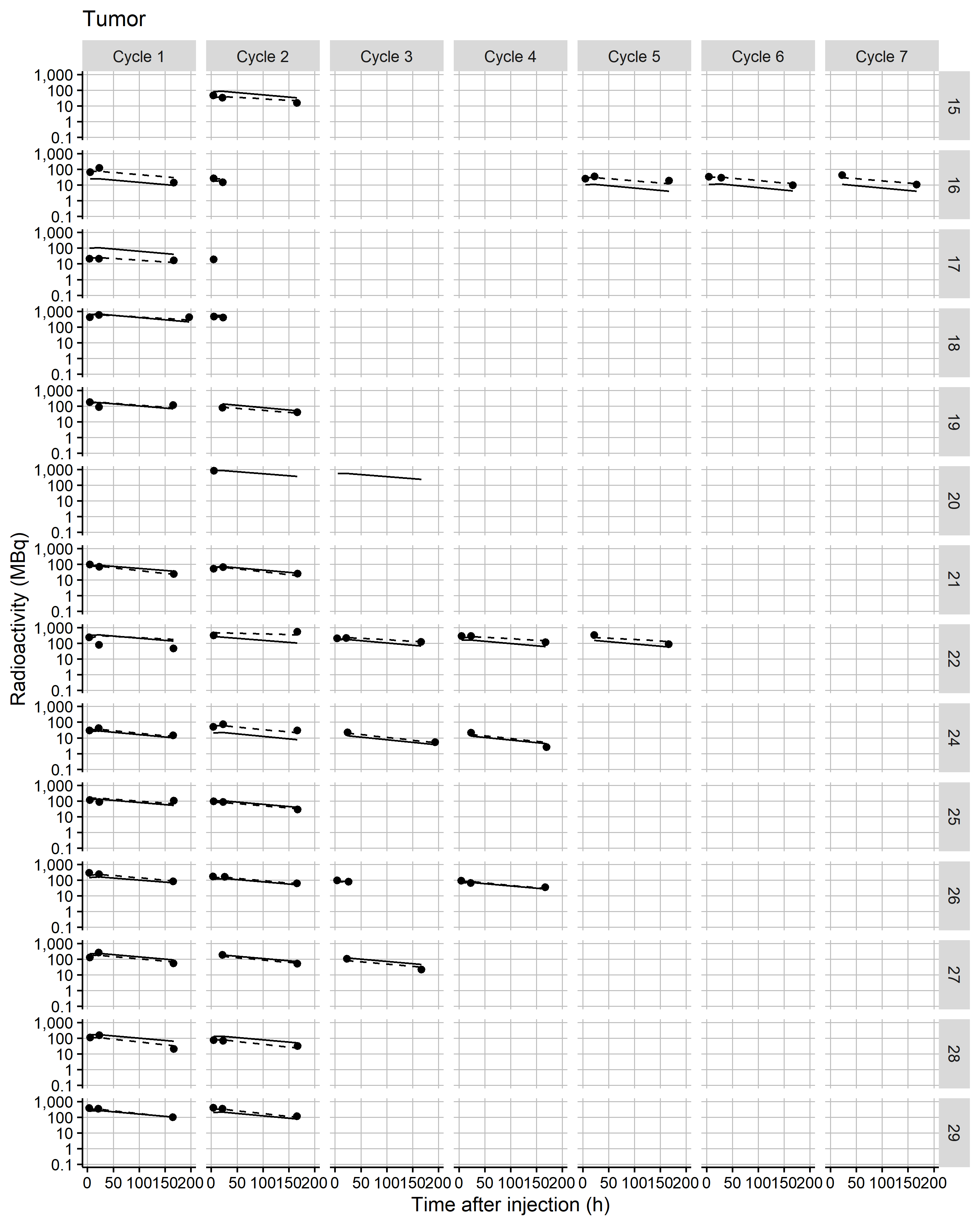

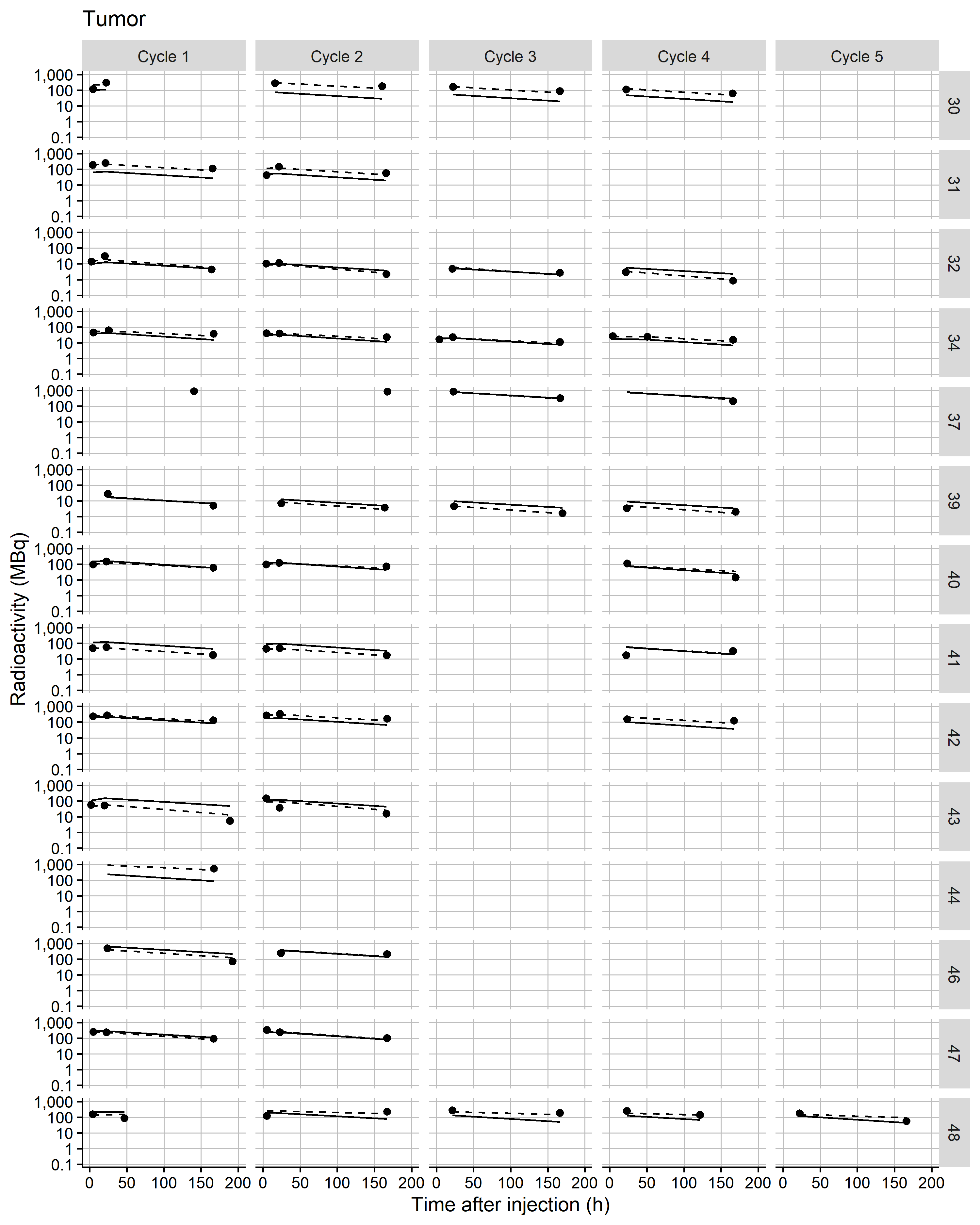

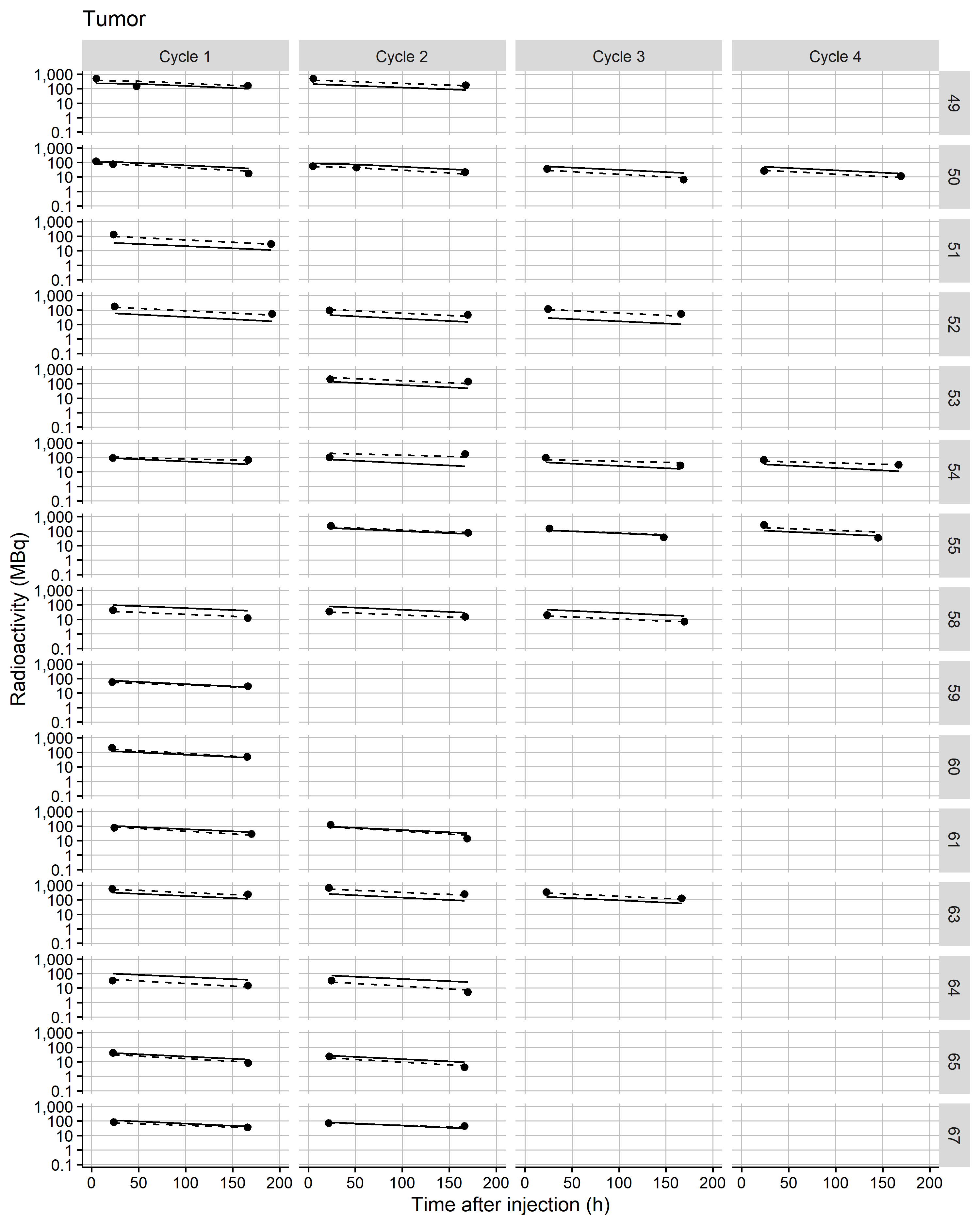

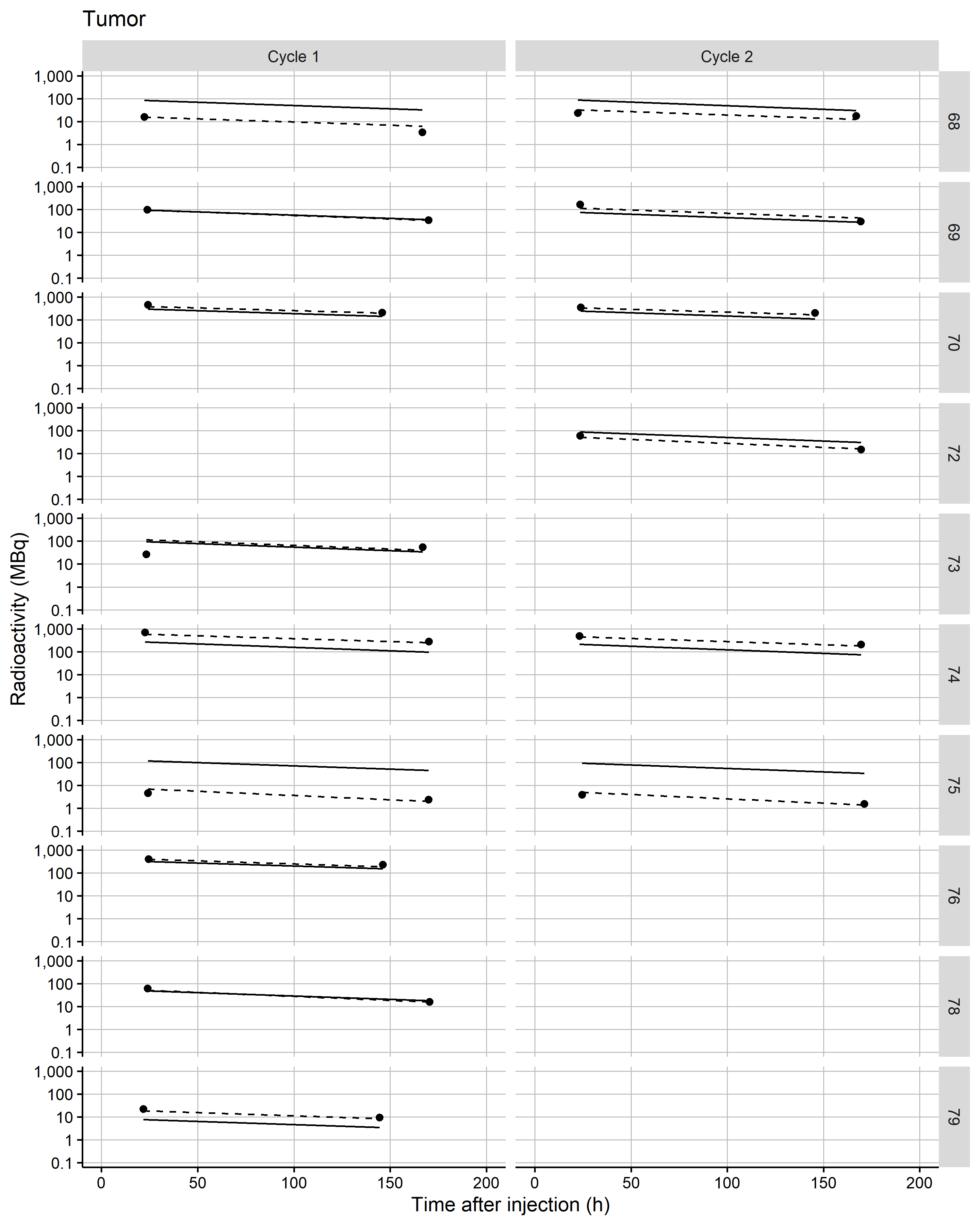
**

**Supplemental Figure 3** – Individual [^177^Lu]Lu-PSMA-I&T concentration over time plots for tumors, where individual (dashed lines) and population (solid lines) predictions based on the final PKPD model as well as observed data (dots) are shown (per cycle).


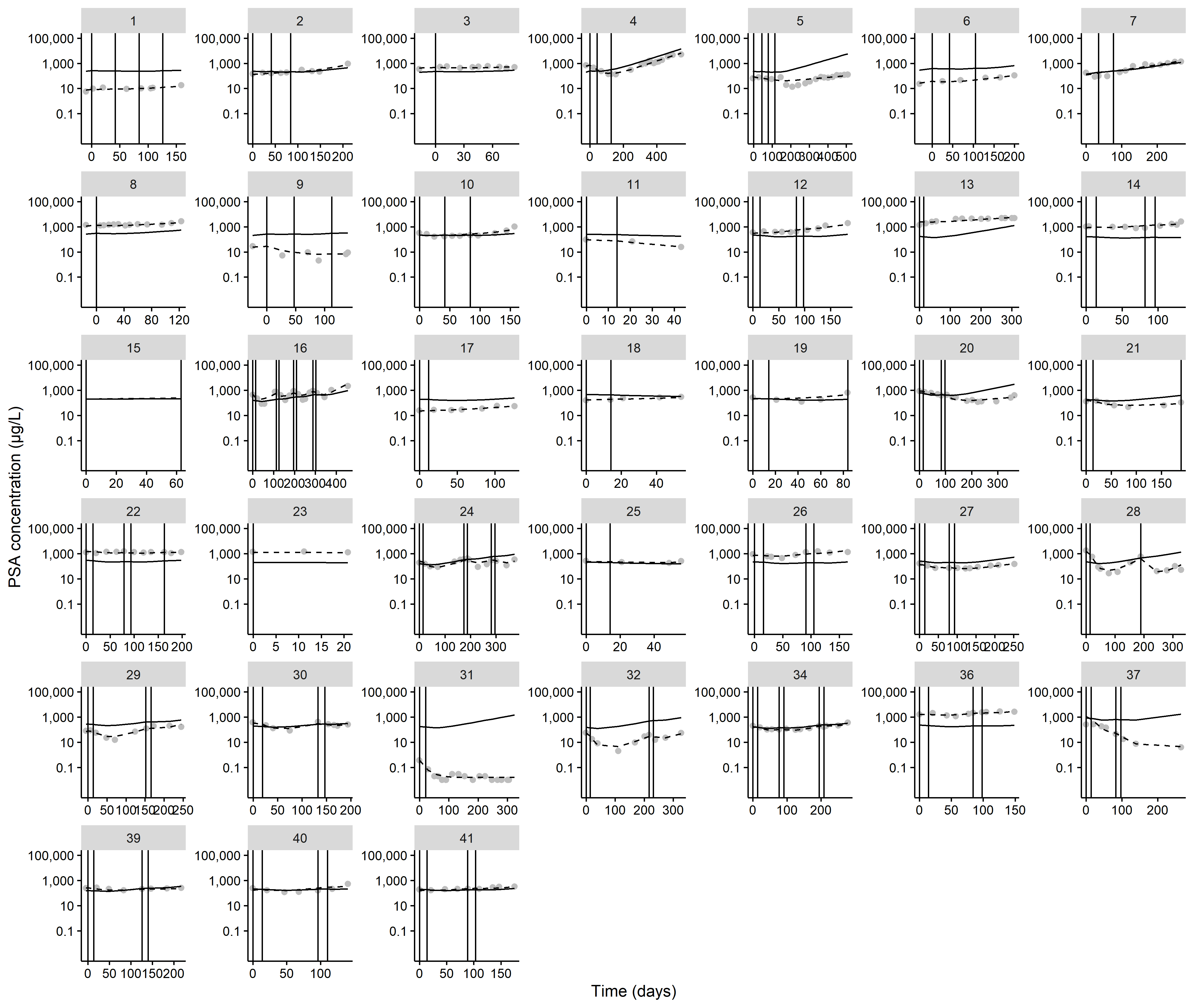

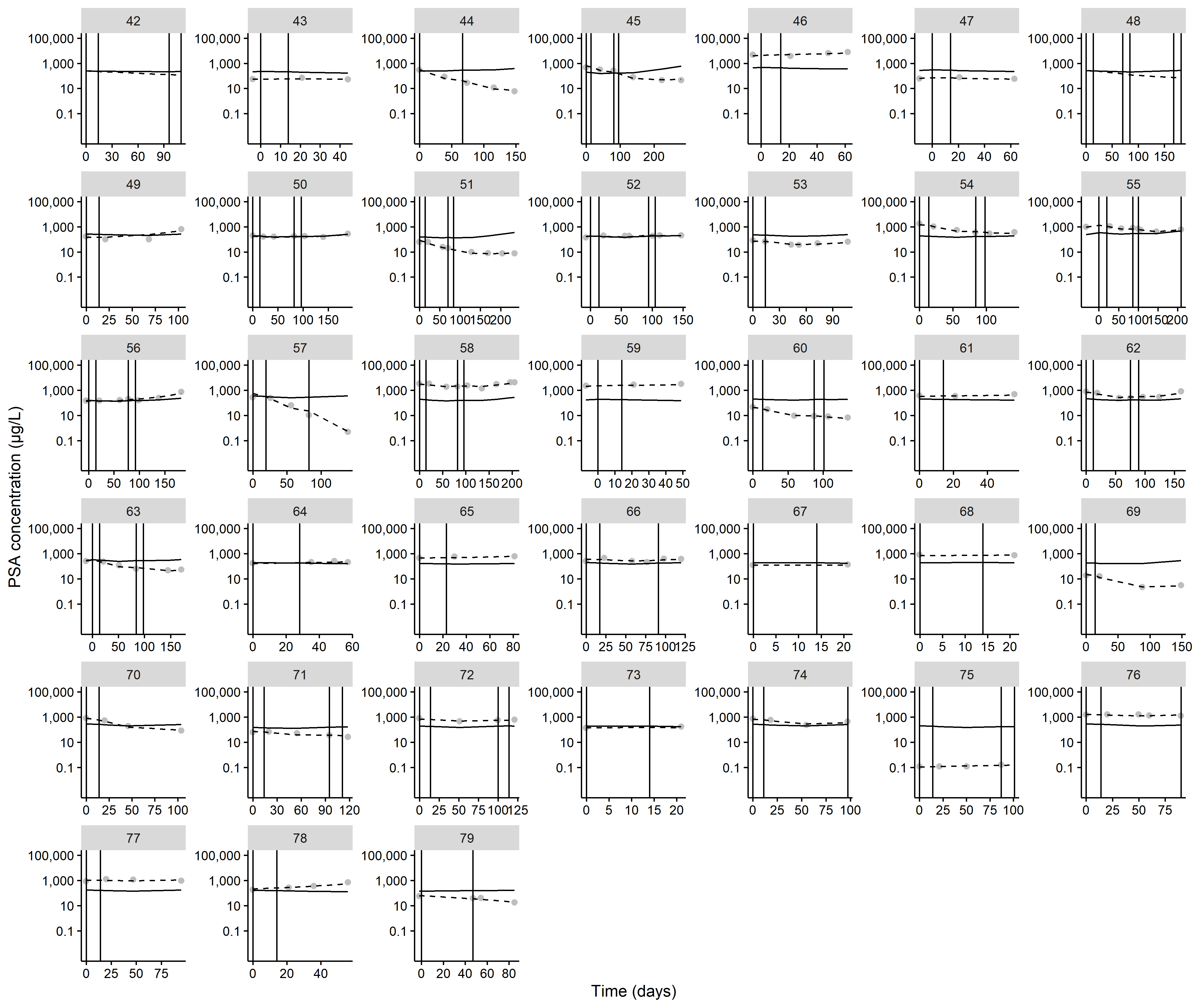


**Supplemental Figure 4** – Individual prostate-specific antigen (PSA) concentrations over time, where vertical lines show [^177^Lu]Lu-PSMA-I&T treatment cycles and individual (dashed lines) and population (solid lines) predictions based on the final PKPD model as well as observed PSA concentrations (dots) are shown.
